# MiR-1224-5p modulates osteogenesis by coordinating osteoblast/osteoclast differentiation via the Rap1 signaling target ADCY2

**DOI:** 10.1038/s12276-022-00799-9

**Published:** 2022-07-13

**Authors:** Liangcong Hu, Xudong Xie, Hang Xue, Tiantian Wang, Adriana C. Panayi, Ze Lin, Yuan Xiong, Faqi Cao, Chengcheng Yan, Lang Chen, Peng Cheng, Kangkang Zha, Yun Sun, Guodong Liu, Chenyan Yu, Yiqiang Hu, Ranyang Tao, Wu Zhou, Bobin Mi, Guohui Liu

**Affiliations:** 1 Department of Orthopaedics, Union Hospital, Tongji Medical College, Huazhong University of Science and Technology, 430022 Wuhan, China; 2 Hubei Province Key Laboratory of Oral and Maxillofacial Development and Regeneration, 430022 Wuhan, China; 3 Department of emergency medicine, Union Hospital, Tongji Medical College, Huazhong University of Science and Technology, 430022 Wuhan, China; 4 Division of Plastic Surgery, Brigham and Women’s Hospital, Harvard Medical School, Boston, MA USA; 5 Department of Neurosurgery, Union Hospital, Tongji Medical College, Huazhong University of Science and Technology, 430022 Wuhan, China; 6 Medical Center of Trauma and War Injuries, Daping Hospital, Army Medical University, 400042 Chongqing, China

**Keywords:** Translational research, Cell signalling

## Abstract

MicroRNAs (miRNAs) broadly regulate normal biological functions of bone and the progression of fracture healing and osteoporosis. Recently, it has been reported that miR-1224-5p in fracture plasma is a potential therapy for osteogenesis. To investigate the roles of miR-1224-5p and the Rap1 signaling pathway in fracture healing and osteoporosis development and progression, we used BMMs, BMSCs, and skull osteoblast precursor cells for in vitro osteogenesis and osteoclastogenesis studies. Osteoblastogenesis and osteoclastogenesis were detected by *ALP, ARS*, and *TRAP* staining and bone slice resorption pit assays. The miR-1224-5p target gene was assessed by siRNA-mediated target gene knockdown and luciferase reporter assays. To explore the Rap1 pathway, we performed high-throughput sequencing, western blotting, RT-PCR, chromatin immunoprecipitation assays and immunohistochemical staining. In vivo, bone healing was judged by the cortical femoral defect, cranial bone defect and femoral fracture models. Progression of osteoporosis was evaluated by an ovariectomy model and an aged osteoporosis model. We discovered that the expression of miR-1224-5p was positively correlated with fracture healing progression. Moreover, in vitro, overexpression of miR-1224-5p slowed Rankl-induced osteoclast differentiation and promoted osteoblast differentiation via the Rap1-signaling pathway by targeting ADCY2. In addition, in vivo overexpression of miR-1224-5p significantly promoted fracture healing and ameliorated the progression of osteoporosis caused by estrogen deficiency or aging. Furthermore, knockdown of miRNA-1224-5p inhibited bone regeneration in mice and accelerated the progression of osteoporosis in elderly mice. Taken together, these results identify miR-1224-5p as a key bone osteogenic regulator, which may be a potential therapeutic target for osteoporosis and fracture nonunion.

## Bone metabolism: A microRNA boosts bone repair and density

A microRNA called miR-1224-5p plays a key role in regulating the balance between bone formation and resorption, and may help in developing therapies for osteoporosis and hard-to-heal fractures. MicroRNAs are small, non-coding RNAs that regulate gene expression. Levels of miR-1224-5p were known to be low in patients with osteoporosis, caused by imbalanced bone resorption and formation, and high in patients with fractures. Guohui Liu and Bobin Mi at Union Hospital, Tongji Medical College, Huazhong University of Science and Technology in Wuhan, China, and coworkers hypothesized that miR-1224-5p might affect the bone resorption/formation balance. They found that miR-1224-5p levels correlated with fracture healing progress. Boosting levels in mice made bones stronger and improved fracture healing, whereas suppressing levels impaired fracture healing and accelerated osteoporosis. These results show that miR-1224-5p represents a potential target for treatment for osteoporosis and for bone-healing deficits.

## Abstract (web summary)

A proposed model illustrating (Supplementary materials e) miRNA-1224-5p-mediated bone cell differentiation. Schematic representation of the mechanism through which miRNA-1224-5p mediates bone cell differentiation in fracture healing and osteoporosis.

## Introduction

Osteoporosis represents an urgent and growing public health problem, is characterized by low bone mass and microstructural destruction of the bone tissue and leads to increased bone fragility and increased susceptibility to fractures^1^. The most common osteoporosis-related fractures are those observed in the hip and vertebrae and result in high levels of morbidity, mortality and nonunion^2^. The epidemiology of hip fractures has highlighted that their rate increases exponentially with age and that females account for three-quarters of cases^1,3^. A recent study calculated that in 2010, there were 2.7 million hip fracture patients, and 51% of these cases were deemed potentially preventable if osteoporosis had been avoided^4^. Current treatments for osteoporosis and nonhealing are comprehensive, including lifestyle management, drug and surgical therapies^5,6^. However, the use of existing antiosteoporosis drugs and surgery can still result in side effects^7^.

Previous studies have reported that miRNAs are involved in the regulation of bone metabolism^8^. For example, miR-212/132 and miR-99b/let-7e/125a negatively regulate TNFAIP3 and IGF1R, IL15 promotes osteoclast differentiation, and miR-23a clusters are regulated by targeting Prdm16, which regulates the transforming growth factor-β-signaling pathway and osteocyte differentiation^9,10^. Recent studies have found that miR-1224 is involved in the regulation of chronic pain via interaction with circRNA-ILIP1I and inhibition of fibroblast proliferation via the TGF-β1/Smad-signaling pathway, thus reducing scar formation^11,12^. In patients with brain trauma, miR-1224 in bone marrow-derived exosomes promoted osteoclast differentiation by targeting the NF-κB signaling pathway and via the Hippo signaling pathway^13,14^. The pathogenesis of osteoporosis and nonunion is due to the imbalance of osteogenesis and osteoclast bone metabolism. The mediation by miR-1224-5p of osteoblast/osteoclast differentiation in patients with osteoporosis and bone fracture has not yet been determined.

MiR-1224-5p is also important in osteoclast effector responses and proliferation^15^. However, the role of miR-1224-5p in osteoporosis and fracture has not been previously reported. Since miR-1224-5p is highly expressed in fracture patients but poorly expressed in osteoporosis patients, we hypothesized that it coordinates the regulation of bone metabolism. To test this supposition, as shown in graphical abstract, we established both fracture and osteoporosis models in ovariectomized and senile animals, revealing the profound impact of miR-1224-5p overexpression on bone fracture healing and the development of osteoporosis and homeostasis.

## Materials and methods

### Animals

All animal experiments were performed in accordance with both the Guidelines of the Intramural Animal Use and Care Committees of Tongji Medical College of Huazhong University of Science and Technology and the ARRIVE guidelines. Eight- and 12-week-old and 18-month-old WT-C57BL/6J female mice were obtained from the Experimental Animal Center of Tongji Medical College of Huazhong University of Science and Technology and housed in pathogen-free facilities under a 12-h light/dark cycle. Animals had ad libitum access to food and water.

### Cell extraction and culture

The bone marrow-derived macrophage (BMM) extraction method was described in a previous study^16^. The cells were cultured in α-MEM with antibiotics (100 mg/ml streptomycin, 100 units/ml penicillin, from Gibco), 10% fetal bovine serum (Gibco), and 30 ng/ml M-CSF (416-ML-050/CF, R&D) and incubated for 12 h in a 37 °C incubator. The adherent cells were removed and resuspended and then cultured for 3 days to obtain BMMs.

Bone marrow mesenchymal stem cells (BMSCs) were extracted according to a previously published method^17,18^.

Skull osteoblast precursor cells were extracted in accordance with the method of Liu et al. ^19^ and cultured in osteoinduction medium (MUBMX-90011, Cyagen Biosciences) to induce differentiation into osteoblasts.

Osteoblasts and osteoclasts were cocultured after seeding BMMs in a Petri dish filled with osteoblasts, and then, 10 nM 1,25-dihydroxyvitamin D_3_, 1 mM prostaglandin E_2_ (1,25(OH)_2_D_3_), and PGE_2_ (all from Sigma) were added, with or without miRNA-1224-5p, and cultured for 5 days. TRAP staining was used to confirm the presence of osteoclasts.

### Cell differentiation

The extracted cells were cultured with BMSC culture medium (MUBMX-90011, Cyagen Biosciences) for 36 h. The culture medium and nonadherent cells were discarded, and the adherent cells represented BMSCs. Third-passage BMSCs were differentiated into adipocytes using adipogenic differentiation medium (MUBMX-90031, Cyagen Biosciences) or into osteoblasts with osteogenic differentiation medium (MUBMX-90021, Cyagen Biosciences).

BMMs underwent induction into osteoclasts in accordance with the method of Liu et al. ^20^. A total of 1*10^5^ BMMs/well were seeded in the wells of a 96-well plate and transfected after 24 h with miR-1224-5p (GenePharma, Shanghai) using Lipo3000 (L3000008, Thermo Science). Osteoclast induction medium (30 ng/ml M-CSF, 50 ng/ml RANKL in α-MEM with 10% FBS, 1× penicillin–streptomycin) was then added and cultured at 37 °C for 4 days, after which osteoclasts were obtained. TRAP reagent was used to confirm that the cells were osteoclasts.

### Apoptosis and proliferation assay

Proliferation was measured in BMSCs, BMMs, and osteoblasts using WST-1 cell proliferation reagent (Roche, Indianapolis, IN). Briefly, for cells treated with miRNA in a 96-well plate, 10 μl of WST-1 was added to each well and incubated for 2 h at 37 °C. Absorption at 450 nm was measured using a microplate reader (Varioskan Flash, Thermo Scientific). The apoptosis of BMMs after treatment with miRNA was measured using an in situ cell death detection kit (Roche) in accordance with the manufacturer’s instructions, the proliferation of BMSCs after treatment with miRNAs was measured by an EDU kit (Thermo Fisher, USA) according with the operating instructions.

### Bone slice resorption pit assay

A total of 1*10^5^ BMMs were seeded onto cortical bone slices (DT-1BON1000-96, Nordic Bioscience Diagnostics, Herlev, Denmark). After 24 h, the BMMs were transfected with miR-1224-5p and then cultured with osteoclast induction medium for 7 days. The slices were then washed repeatedly with PBS to remove adherent cells, after which the bone resorption pits were observed by scanning electron microscopy.

### Assessment of bone mineralization capability

BMSCs and primary calvarial osteoblasts (Pre-Ob) were transfected with different miRNAs, and the cells were cultured in the corresponding osteogenic induction medium (BMSCs: MUBMX-90021, Pre-Ob: MUBMX-90011, Cyagen Biosciences) to induce osteoblast differentiation. A BCIP/NBT alkaline phosphatase kit (C3206, Beyotime Biotechnology) was used to identify ALP, and an alkaline phosphatase detection kit (P0321S, Beyotime Biotechnology) was used to measure the ALP concentration. An osteogenesis assay kit (C0148S, Beyotime Biotechnology) was used to identify bone mineralization nodules according to a protool provided by the manufacturer.

### SiRNA-mediated target gene knockdown

Mouse ADCY2 and control small interfering RNAs (siRNAs) were purchased from Genechem (Shanghai, China). Each siRNA consisted of pools with at least three target-specific 25-nucleotide siRNAs designed to silence target gene expression. The siRNA was transfected into BMMs using Lipo3000 (Invitrogen) to silence the target genes, in accordance with the supplier’s instructions.

### Luciferase reporter assay

A luciferase reporter assay was performed as described previously without modifications^21^. The 3’-UTR of mouse ADCY2, which contains the miR-1224-5p binding sequence, was amplified using PCR from murine genomic DNA and subcloned into a pGL3 vector (E1741; Promega, USA). A Hieff Mut™ site-directed mutagenesis kit (11003ES10; Yeasen, China) was used to mutate this sequence.

### High-throughput sequencing and RT-PCR

The mRNA expression levels in mouse BMMs were quantified using high-throughput sequencing 24 h after stimulation in the presence of 30 ng ml^−1^ M-CSF and 50 ng ml^−1^ RANKL with or without agomiR-1224-5p. After we received ethical approval from the hospital and signed informed consent from each patient, 5 ml of serum was collected from peripheral blood on the third day following femoral fracture (*N* = 3), and nonfracture patients (*N* = 3) were also analyzed as the normal group. Total RNA was isolated using an RNAqueous^®^-Midi Kit (AM1911, Thermo Fisher) in accordance with the manufacturer’s instructions.

High-throughput sequencing was performed in triplicate using an Illumina HiSeq X Ten Second Generation Sequencing Platform (SeqHealth Co., Wuhan, China) to analyze the differentially expressed miRNAs (DEMs). DEMs that were significantly distinct between the two groups were identified using volcano plot filtering and fold change filtering to identify DEMs in the two samples. Hierarchical cluster analysis was conducted using hiplot online software (https://hiplot.com.cn/basic). A 2-fold difference in the expression threshold, with a *P* value of <0.01, was selected for the identification of differentially expressed transcripts (sequencing different genes and different miRNAs with significantly upregulated expression are shown in Supplementary Materials i and j).

For RT-PCR, cDNA was prepared from 1 mg RNA using Reverse Transcription 10X Buffer (A3561, Promega) and analyzed with SYBR Green Master Mix (SABiosciences) using a Real-Time PCR system (CFX-Connect, Bio-Rad). Primers were designed (PrimerBank, https://pga.mgh.harvard.edu/primerbank/) for each targeted gene, as detailed in Table 1. Relative transcript expression was calculated using the 2^−ΔΔCt^ method after normalization against GAPDH. Expression data are presented as a fold increase relative to the endogenous control.

**Table 1:** mRNA and miRNA primer sequences.

MicroRNA or gene name	Primer sequence (5’–3’)
mmu-miRNA-1224-5p-forward	5’-GCGGCGGGTGAGGACTGGGGAG-3'
mmu-miRNA-1224-5p-reverse	5’-ATCCAGTGCAGGGTCCGAGG-3'
hsa-miRNA-1224-5p-forward	5’-GCGGCGGGTGAGGACTCGGGAG-3'
hsa-miRNA-1224-5p-reverse	5’-ATCCAGTGCAGGGTCCGAGG-3'
hsa-ALP-forward	5’-ACTGGGGCCTGAGATACCC-3'
hsa-ALP-reverse	5’-TCGTGTTGCACTGGTTAAAGC-3'
mmu-ALP-forward	5’-GGCAAAGAGGGAGCTAGAA-3'
mmu-ALP-reverse	5’-ATGGCCGTGCAGATGTA-3'
hsa-COL1A1-forward	5’-GAGGGCCAAGACGAAGACATC-3'
hsa-COL1A1-reverse	5’-CAGATCACGTCATCGCACAAC-3'
mmu-COL1A1-forward	5’-CAGAGGCGAAGGCAACA-3'
mmu-COL1A1-reverse	5’-GTCCAAGGGAGCCACATC-3'
hsa-OCN-forward	5’-GGCGCTACCTGTATCAATGG-3'
hsa-OCN-reverse	5’-GTGGTCAGCCAACTCGTCA-3'
mmu-OCN-forward	5’-GCTGTTTGTTCGGGTCTC-3'
mmu-OCN-reverse	5’-GGGCCAAAGTCAGCATC-3'
hsa-RUNX2-forward	5’-TGGTTACTGTCATGGCGGGTA-3'
hsa-RUNX2-reverse	5’-TCTCAGATCGTTGAACCTTGCTA-3'
mmu-RUNX2-forward	5’-GCCGGGAATGATGAGAAC-3'
mmu-RUNX2-reverse	5’-TGGGGAGGATTTGTGAAGA-3'
mmu-GAPDH-forward	5’-AGGTCGGTGTGAACGGATTTG-3'
mmu-GAPDH-reverse	5’-GGGGTCGTTGATGGCAACA-3'
hsa-GAPDH-forward	5’-GGAGCGAGATCCCTCCAAAAT -3'
hsa-GAPDH-reverse	5’-GGCTGTTGTCATACTTCTCATGG-3'
mmu-ACP5-forward	5’-CACTCCCACCCTGAGATTTGT-3'
mmu-ACP5-reverse	5’-CATCGTCTGCACGGTTCTG-3'
hsa-ACP5-forward	5’-GACTGTGCAGATCCTGGGTG-3'
hsa-ACP5-reverse	5’-GGTCAGAGAATACGTCCTCAAAG-3'
mmu-c-fos-forward	5’-CGGGTTTCAACGCCGACTA-3'
mmu-c-fos-reverse	5’-TTGGCACTAGAGACGGACAGA-3'
hsa-c-fos-forward	5’-CCGGGGATAGCCTCTCTTACT-3'
hsa-c-fos-reverse	5’-CCGGGGATAGCCTCTCTTACT-3'
mmu-NFACT1-forward	5’-GGAGAGTCCGAG AATCGAGAT-3'
mmu-NFACT1-reverse	5’-TTGCAGCTAGGAAGTACGTCT-3'
hsa-NFACT1-forward	5’-GCAGAGCACGGACAGCTATC-3'
hsa-NFACT1-reverse	5’-GGGCTTTCTCCACGAAAATGA-3'
mmu-CTSK-forward	5’-GAAGAAGACTCACCAGAAGCAG-3'
mmu-CTSK-reverse	5’-TCCAGGTTATGGGCAGAGATT-3'
hsa-CTSK-forward	5’-ACACCCACTGGGAGCTATG-3'
hsa-CTSK-reverse	5’-GACAGGGGTACTTTGAGTCCA-3'
mmu-SRC-forward	5’-GAACCCGAGAGGGACCTTC-3'
mmu-SRC-reverse	5’-GAGGCAGTAGGCACCTTTTGT-3'
hsa-SRC-forward	5’-GAGCGGCTCCAGATTGTCAA-3'
hsa-SRC-reverse	5’-CTGGGGATGTAGCCTGTCTGT-3'

### Western blot

In short, protein samples were boiled for 10 min at 95 °C, electrophoresed in a 12% SDS polyacrylamide gel, transferred to PVDF transfer membranes (88520, Thermo Fisher Scientific) and blocked with fast blocking solution (PS108, Epizyme Biotech) at room temperature for 20 min. The samples were probed with an aliquot of primary antibody (Table 2) and incubated overnight at 4 °C. After the membrane was washed with TBST at room temperature, secondary antibody (LF101, Epizyme Biotech) was added and incubated. An Omni-ECL™ ultrasensitive chemiluminescence detection kit (dSQ201, Epizyme Biotech) was used to develop the protein banding. A ChemiDoc-It^®^ 610 imaging system (P/N 97-0270-02, Ultra-Violet Products, Ltd., Cambridge) was used to display the Western blots. ImageJ software was used to analyze the gray intensity levels of the protein bands. Quantification was achieved by normalization to the loading control.

**Table 2:** Antibody listing.

Antibody	Vendor details
Mouse anti-ADCY2	Affinity Biosciences Cat# DF2270,RRID: AB_2839499
Mouse anti-Rap1	Affinity Biosciences Cat# DF8598, RRID:AB_2841802
Mouse anti-Rac1/cdc42	Affinity Biosciences Cat# DF6322, RRID:AB_2838287
Mouse anti-p-Rac1/cdc42	Affinity Biosciences Cat# AF2393, RRID:AB_2845407
Mouse anti-Rho-A	Affinity Biosciences Cat# AF6352, RRID:AB_2835157
Mouse anti-p-Rho-A	Affinity Biosciences Cat# AF3352, RRID:AB_2834767
Mouse anti-Rac1/2/3	Affinity Biosciences Cat# AF9178, RRID:AB_2843368
Mouse anti-FAK	Affinity Biosciences Cat# AF6397, RRID:AB_2835232
Mouse anti-p-FAK	Affinity Biosciences Cat# AF3398, RRID: AB_2834829
Mouse anti-NFATc1	MA3-024, Thermo Fisher

### Chromatin immunoprecipitation assay

ChIP assays were performed using a simple ChIP assay kit (Cell Signaling Technology) in accordance with the manufacturer’s protocol using the following antibodies (the antibodies are shown in Table 3). The precipitated DNA was quantified using real-time PCR (CFX-Connect, Bio-Rad). Data are presented as the percentage of input DNA (the ChIP primers are shown in Table 4).

**Table 3:** ChIP antibody listing.

Antibody	Vendor details
Acetylated histone H4	H4ac, #39926, Active Motif
ADCY2	Affinity Biosciences Cat# DF2270, RRID: AB_2839499
Rap1	Affinity Biosciences Cat# DF8598, RRID: AB_2841802

**Table 4:** ChIP primers.

Nfatc1-forward	GACTTTGTGTCCCCAGGAGG
Nfatc1 -reverse	GCAGAGGCAGAACACTTCCA
Nfatc1 negative -forward (20 kb down)	GTGCTGTGTGTTATGGCACC
Nfatc1 negative -reverse (20 kb down)	CCTGGGGGCAGTGTTTACAG
Ocn-forward	CTGAGCACATGACCCCCAAT
Ocn-reverse	TCGGCTACTCTGTGCTCTCT
Ocn negative-forward (2 kb down)	AGAAGGCAGGTGTGTTGAGG
Ocn negative-reverse (2 kb down)	TGCTCTTCCTGCCTCTACCT

### Immunohistochemical staining

After isolation, the femurs were fixed overnight in 4% formaldehyde. The bones were decalcified in EDTA (AR1071, Boster Biological Technology) at room temperature for 20 days and then paraffin-embedded (BMJ-A, Changzhou Zhongwei Electronic Instrument Co., Ltd.). The paraffin blocks were then sliced (HM325, Thermo Fisher), after which the sections were treated in sodium citrate buffer (10 mM sodium citrate, 0.05% Tween 20, pH 6.0) at 90 °C for 20 min to recover the antigens. The samples were then incubated with antibody overnight at 4 °C. Positive detection was achieved by staining with Dako EnVision™+ System/HRP with Mo (DAB+) and K400711-2. The optical density (IOD) of positively stained antigens was quantified using ImageJ software and normalized by stained area.

### Animal model

Male and female 8- and 12-week-old and 18-month-old C57BL/6J mice were used in the experimental models. Animals were anesthetized with intraperitoneal administration of 1% sodium pentobarbital (0.1 ml/10 g body weight). After surgery, the animals received an intraperitoneal injection of 5% glucose (0.1 ml/10 g body weight) to promote resuscitation. A 10 ml suspension of ibuprofen (Meilin, Shanghai Johnson Pharmaceutical Co., Ltd.) was added to 500 ml of drinking water to relieve postoperative pain. The surgical site was closed using 4.0 absorbable sutures, and 5.0 absorbable sutures were used for ligature. A cortical bone defect model was established in accordance with published surgical protocols^20,22^. A cranial bone defect model was established in accordance with published surgical protocols^23,24^. A femoral fracture model was established in accordance with published protocols^25^. An ovariectomy model was established in accordance with published protocols^26^.

### Microcomputed tomography (μCT) analysis

Microcomputed tomography (μCT) analysis was performed as described previously^25^. Femurs were scanned using a Bruker SkyScan 1176 μCT (2400 frames, 5 frames/frame, 37 kV, 121 mA). Bruker μCT analysis software (NReconServer, CT An, 3D model, CT Vol, DataViewer, Bruker, Germany) was used to quantify bone formation parameters: BV/TV (bone volume/tissue volume) per tissue volume, BMD (bone mineral density), BS/BV (bone surface/bone volume), Tb.N (trabecular number), Ct.Th (average cortical thickness), cortical bone area (Ct.Ar), Tb.Sp (trabecular separation/spacing), and Tb.Th (trabecular separation/trabecular thickness).

### Statistical analysis

Differences between two groups were compared using Student’s *t* test, with *P* < 0.05 considered statistically significant. Multiple comparisons were performed by one-way analysis of variance followed by Tukey’s test. All statistical analyses were performed using GraphPad Prism 7 and SPSS v22.0 software. All data are expressed as the mean ± s.d., *P < 0.05, ***P < 0.01, #*P* > 0.05.

## Results

### MiRNA-1224-5p accelerates bone healing via enhancement of BMSC differentiation to osteoblasts

**Fig. 1:**
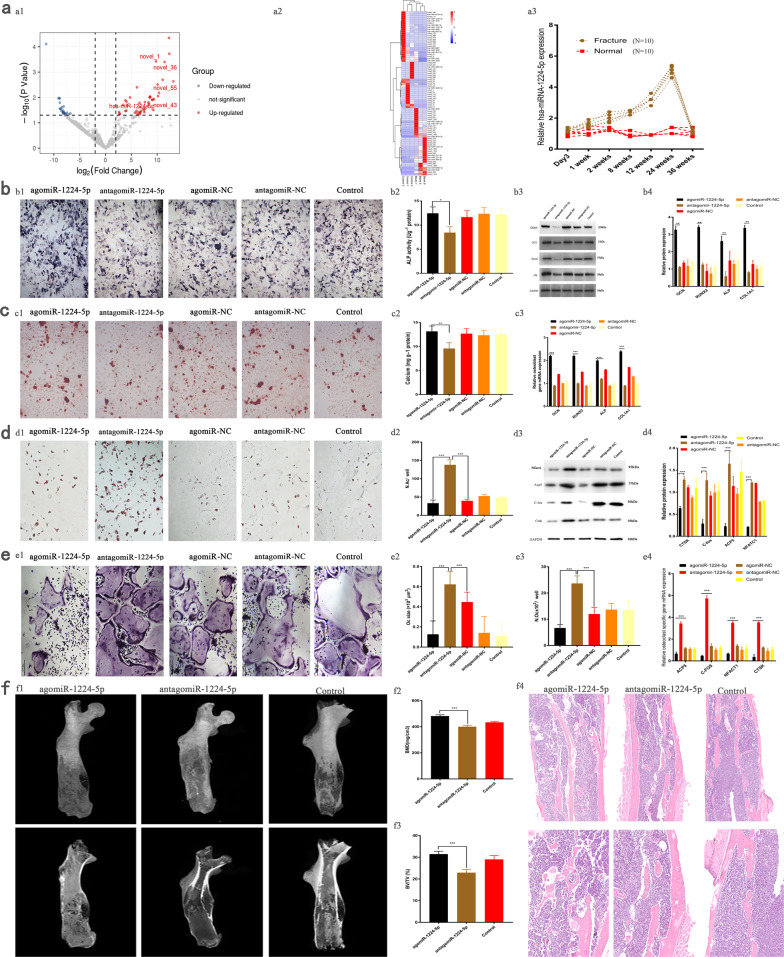
MiRNA-1224-5p accelerates bone repair by enhancing BMSC differentiation to osteoblasts. **a1** The results of peripheral blood sequencing of patients showed that the expression of miR-1224-5p in the fracture group was significantly higher than that in the normal group (control patients vs. fracture patients = 3 vs. 3). **a2** The differentially expressed miRNA heatmap shows the top 85 miRNAs with upregulated expression in the peripheral blood of the fracture group compared with those of the control patients (*P* < 0.001, log2FC is greater than or equal to 2). **a3** The trend of miR-1224-5p expression in the peripheral blood of patients after fracture is consistent with the bone remodeling process of fracture healing. **b1**, **b2** Alkaline phosphatase (ALP) staining showed that BMSCs were induced to osteoblasts for 2 weeks after miRNA transfection, and ALP staining in the agomR-1224-5p group was significantly enhanced. **b3**, **b4** In the agomiR-1224-5p group, the expression of key osteogenic target proteins and mRNAs was significantly upregulated compared with that in the other groups. Scale bar, 500 µm. **c1**, **c2** Alizarin red (ARS) staining results showed that BMSCs were induced to osteoblasts for 3 weeks after transfection, and the mineralized nodules in the agomR-1224-5p group were significantly enhanced. **c3** The expression of key osteogenic target mRNAs in the agomiR-1224-5p treatment group was significantly upregulated compared with that in the other groups. Scale bar, 500 µm. **d1**, **d2** The results of oil red O staining showed that BMSCs were induced to differentiate into adipocytes for 3 weeks after transfection, and the ability of agomR-1224-5p to form fat was significantly weakened. **e1**–**4** After BMMs were transfected with miRNA and induced to differentiate into osteoclasts for 5 days, the number and size of osteoclasts in the agomiR-1224-5 group were significantly smaller than those in the other groups. **d3**, **d4** The expression levels of osteoclast differentiation marker proteins and mRNAs in the agomiR-1224-5p group were lower than those in the other groups. **f1**–**3** In the agomR-1224-5p group, BV/TV and BMD were higher than those in the control group and the antagomiR-1224-5p group. **f4** The results of the local H&E sections of the fracture model showed that the continuity of the new callus at the fracture end of the agomiR-1224-5p group was better than that of the control group and the other groups. The results are presented as the mean ± standard deviation, *N* = 5; ^#^*P* ≥ 0.05; *P < 0.05, **P < 0.01; ***P < 0.001 by analysis of variance (ANOVA) with Tukey’s post-hoc test.

After we received ethical approval from the hospital and signed informed consent from each patient, 5 ml of serum was collected from peripheral blood on the third day following femoral fracture, and nonfracture patients were also analyzed as the normal group. MiRNAs were analyzed using an Illumina HiSeq X Ten Second Generation sequencing platform (SeqHealth Co., Wuhan, China). The results demonstrated that of 78 differentially expressed miRNAs, 46 had upregulated expression, and 32 had downregulated expression. Among these, miR-1224-5p had significantly upregulated expression (Fig. 1a1, 2). Serum miR-1224-5p expression in the fracture group was significantly higher than that in the normal group on day 3 and weeks 1, 2, 8, 12, 24, and 36 following fracture. The expression of miR-1224-5p was correlated with fracture healing (Fig. 1a3 and Supplementary Fig. 3 and Supplementary Table 1, *N* = 10). Thus, we speculate that the expression of miR-1224-5p may be related to the capability of bone marrow mesenchymal stem cells (BMSCs) to form bone. To confirm this hypothesis, we transfected human BMSCs with miR-1224-5p, and then, osteogenesis and adipogenesis were induced. It is apparent that miR-1224-5p can promote the differentiation of BMSCs into osteoblasts (Fig. 1b1–4 and 1c1–3) and inhibit the differentiation of BMSCs into adipocytes (Fig. 1d1, 2). Because bone formation is closely related to the balance of osteoblast and osteoclast activity^27^, human bone marrow-derived macrophages (BMMs) were transfected with miR-1224-5p, and their effect on BMM differentiation into osteoclasts was analyzed. The results indicate that miR-1224-5p significantly inhibited the differentiation of BMMs into osteoclasts (Fig. 1e1–4 and 1d3, 4).

Eight-week-old C57/BL6J male mice were then used to establish a femoral fracture model, and miRNAs were locally administered, in accordance with a previously described method^25^. The results demonstrate that agomiR-1224-5p significantly promoted fracture healing (Fig. 1f1–4). Using this model, we found that the expression of miR-1224-5p increased in the peripheral blood of fracture patients. Elevated miR-1224-5p promoted the differentiation of BMSCs into osteoblasts and inhibited the differentiation of BMMs into osteoclasts. However, the specific mechanism remains unclear, and the effect of miR-1224-5p on changes in bfione mass in adult and elderly mice still requires clarification.

### Overexpression of miR-1224-5p increases bone mass in young adult mice

**Fig. 2:**
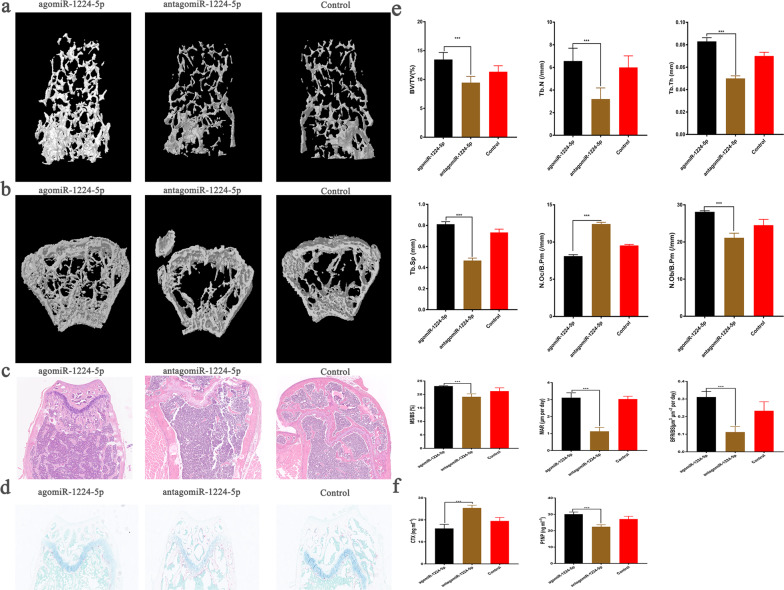
Overexpression of miR-1224-5p increases bone mass in young adult mice. **a**, **b** Micro-CT reconstruction of the representative distal femur. The trabeculae of the agomiRNA-1224 group were significantly increased. **c** HE-stained decalcified distal femur section. The bone trabeculae of the agomiRNA-1224 group were significantly thicker, ordered, and increased. **d** TRAP staining of distal femoral sections. The TRAP-positive cells in the agomiRNA-1224 group were significantly fewer than those in the control group and the antagomiRNA-1224 group. **e** Morphological analysis of the femoral metaphysis. **f** Serum CTX and P1NP expression levels. The results are presented as the mean ± standard deviation, *N* = 5; ^#^*P* ≥ 0.05; *P < 0.05, **P < 0.01; ***P < 0.001 by analysis of variance (ANOVA) with Tukey’s post-hoc test. Scale bar, 500 µm.

AgomiR-1224-5p (15 μg/10 g body weight, once every three days; Gema, Shanghai, China), antagomiR-1224-5p, or PBS was injected into the tail vein of 9-week-old mice for 6 weeks to investigate the role of miR-1224-5p during bone remodeling. Microcomputed tomography (μCT) scan analysis of the secondary spongiosa of the distal femoral metaphysis showed that the trabecular bone volume (BV/TV) of mice in the agomiR-1224-5p group was significantly greater than that of the control and antagomiR-1224-5p groups (Fig. 2a, b, d). H&E staining further confirmed the presence of enhanced trabecular bone in the agomiR-1224-5p group (Fig. 2c). Histomorphological analysis indicated that the number of osteoclasts in the agomiR-1224-5p group was significantly lower (N.Oc/B.Pm), and the number of osteoblasts was significantly higher (N.Ob/B.Pm; Fig. 2e). In addition, the mineralization deposition rate (MAR) and rate of bone formation (BFR/BS) in the agomiR-1224-5p group were significantly higher than those in the other two groups (Fig. 2e). Furthermore, enzyme-linked immunosorbent assay (ELISA) analysis of serum bone transformation markers demonstrated that the levels of the bone resorption marker serum C-terminal telopeptides of collagen type I (CTX) was significantly downregulated and that of the bone formation marker P1NP was significantly upregulated in the agomiR-1224-5p group compared with the other groups (Fig. 2f).

### Overexpression of miR-1224-5p leads to bone formation in elderly mice

**Fig. 3:**
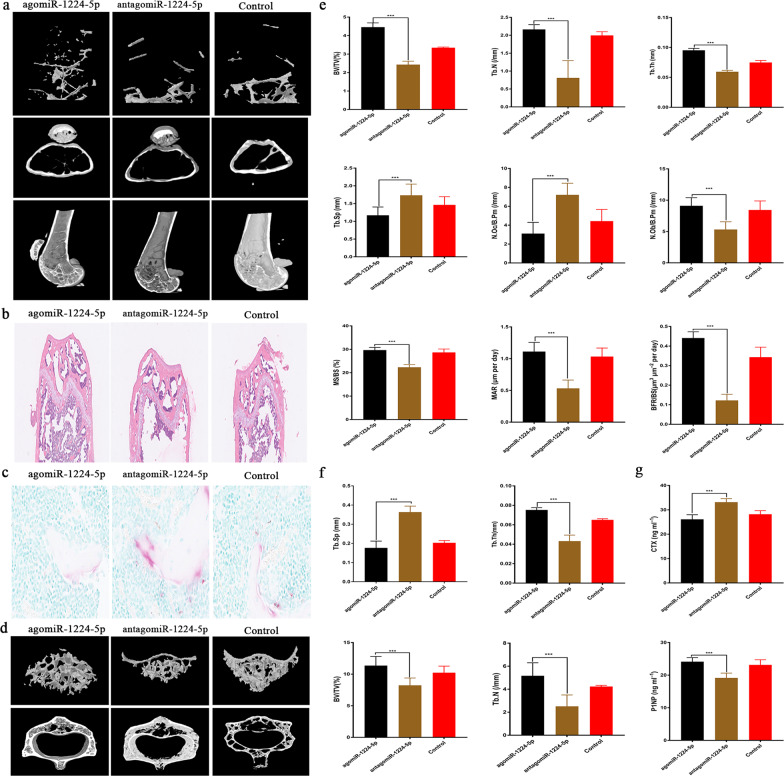
Overexpression of miR-1224-5p leads to bone formation in elderly adult mice. **a** MicroCT reconstruction of the representative distal femur. The trabeculae of the agomiRNA-1224 group were significantly increased. **b** H&E-stained decalcified distal femur section. The bone trabeculae of the agomiRNA-1224 group were significantly thicker, ordered, and increased. **c** TRAP staining of distal femoral sections. The TRAP-positive cells in the agomiRNA-1224 group were significantly fewer than those in the control group and the antagomiRNA-1224 group. **d** MicroCT reconstruction of the representative (4th lumbar) L4 vertebral body. The trabeculae of the agomiRNA-1224 group were significantly increased. **e** Morphological analysis of the femoral metaphysis. **f** Analysis of morphological results of vertebral body tissue. **g** Serum CTX and P1NP expression levels. The results are presented as the mean ± standard deviation, *N* = 5; ^#^*P* ≥ 0.05; *P < 0.05, **P < 0.01; ***P < 0.001 by analysis of variance (ANOVA) with Tukey’s post-hoc test. Scale bar, 500 µm.

To explore the function of miR-1224-5p in pathological conditions of aging, we intraperitoneally injected agomiRNA-1224-5p (15 μg/10 g body weight, once every 3 days, GenePharma, Shanghai, China), antagomiRNA-1224-5p, or PBS into 18-month-old mice for 6 weeks. Consistent with the results observed in young adult mice, agomiR-1224-5p treatment caused increased trabecular bone in the metaphysis of old mice, while the number of both osteoclasts (N.Oc/B.Pm) and osteoblasts (N.Ob/B.Pm) decreased (Fig. 3a, c, e). μCT image reconstruction of the femoral metaphysis and 4th lumbar vertebrae demonstrated significantly increased trabecular bone, consistent with that in young mice (Fig. 3d, f). In addition, the expression of the bone resorption marker CTX in plasma was significantly downregulated, and the expression of the bone formation marker P1NP was significantly higher in the agomiR-1224-5p group (Fig. 3g).

### MiR-1224-5p inhibits osteoclastogenesis and promotes osteoblastogenesis

**Fig. 4:**
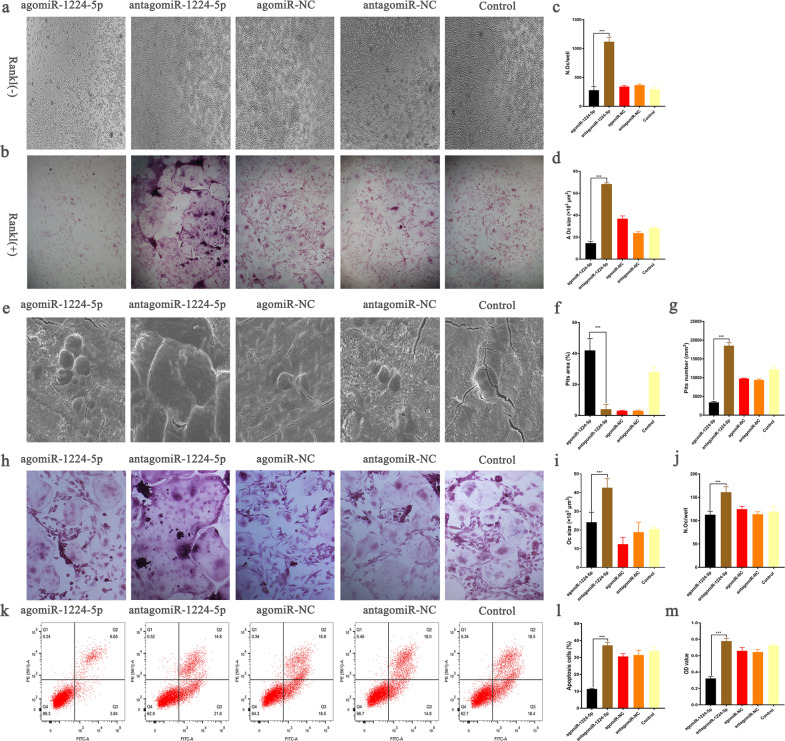
MiR-1224-5p inhibits RANKL-induced osteoclastogenesis. **a**, **b** TRAP staining pictures of representative BMMs 5 days after osteoclast differentiation. **c**, **d** The number and area of bone resorption pits. **e** The results of the representative bone resorption pit experiment on bovine bone slices after 9 days of culture. **f**, **g** Area and number of absorption pits. **h** A representative picture of osteoclasts and osteoblasts cocultured for 5 days in medium containing 1,25(OH)2D3 and PGE2 after transfection with different miRNAs. **i**, **j** Number and size of osteoclasts. **k**–**m** Cell proliferation and apoptosis experiments. BMMs were transfected with different miRNAs for 6 h and cultured for 48 h with 30 ng/ml M-CSF and 50 ng/ml RANKL. The results are presented as the mean ± standard deviation, *N* = 5, scale bar, 500 µm.

The direct effect of agomiR-1224-5p on osteoclast differentiation was then evaluated. First, osteoclast differentiation experiments were conducted in vitro, and the results indicated that miR-1224-5p alone could not induce bone marrow-derived macrophages to differentiate into osteoclasts (Fig. 4a). We then supplemented the culture medium with receptor activator of nuclear factor kappa-B ligand (RNAKL). AgomiR-1224-5p treatment significantly inhibited osteoclast differentiation compared with that of the blank and antagomiR-1224-5p groups. The number of tartrate-resistant acid phosphatase (TRAP)-positive multinucleated cells decreased significantly by day 5 after agomiR-1224-5p treatment (Fig. 4a, b). In addition, following treatment with antagomiR-1224-5p, large osteoclasts with large cytoplasmic components were formed, indicating indirectly that agomiR-1224-5p may prevent osteoclast maturation (Fig. 4b, d). In contrast, during the 5-day period of induction, only a few small osteoclasts were formed in the agomiR-1224-5p group (Fig. 4b, d). Furthermore, a bovine bone slice absorption pit assay was used to evaluate the effect of agomiR-1224-5p on bone resorption by osteoclasts. The results indicated that treatment with agomiR-1224-5p resulted in fewer absorption pits and fewer osteoclasts than those in the control and antagomiR-1224-5p groups (Fig. 4e, g). Finally, BMMs were cocultured with skull-derived osteoblasts to simulate an in vivo environment and enumerate the osteoclasts formed. The results showed that treatment with agomiR-1224-5p decreased the number and nuclear size of TRAP-positive cells (Fig. 4h, j).

Furthermore, to explore whether agomiR-1224-5p can modify the phenotype and number of BMSCs, 6 weeks after young adult and elderly mice were treated by tail vein injection, we analyzed extracted BMSCs by flow cytometry. The results demonstrated that the number of BMSCs that had undergone apoptosis was significantly lower in the agomiR-1224-5p group than in the other two groups (Fig. 4k, m).

Next, we assessed the effect of miRNA-1224-5p on BMSCs proliferation by the EDU staining. However, there was no significant difference in proliferation rate between three groups (Supplementary Fig. 4a, b).

Moreover, the effect of miR-1224-5p on osteoblast differentiation was investigated in vitro. The results demonstrate that agomiR-1224-5p significantly promoted the differentiation of BMSCs into osteoblasts and accelerated the differentiation of osteoblast precursor cells into osteoblasts (Supplementary Fig. 1a, h).

We also investigated whether miR-1224-5p displayed a similar regulatory role in osteoblast differentiation via the Rap1 signaling pathway. Western blot analysis showed that agomiR-1224-5p suppressed the phosphorylation of p-Rho-A in osteoblast precursor cells (Supplementary Fig. 1i). In addition, a ChIP assay confirmed that agomiRNA-1224-5p significantly increased the abundance of acetylation (H4ac) at the binding site of histone H4 and the osteocalcin (OCN) promoter (Supplementary Fig. 1j). Because the activation of Rac1 could hinder the activation of the BMP-signaling pathway, which is critical for osteoblast differentiation^28^, we explored the influence of agomiR-1224-5p on the BMP signaling pathway. Interestingly, agomiR-1224-5p attenuated the induction of Rac1 phosphorylation by BMP2 (100 ng/ml, 355-BM-050/CF, R&D) or 10% fetal bovine serum (FBS; Gibco) in osteoblasts (Supplementary Fig. 1k, l). The evidence above suggests that agomiR-1224-5p is dependent on Rap1 and ADCY2 to increase the transcription of BMP1 to stimulate osteoblast generation.

### MiRNA-1224-5p inactivates ADCY2-dependent Rap1 signaling

**Fig. 5:**
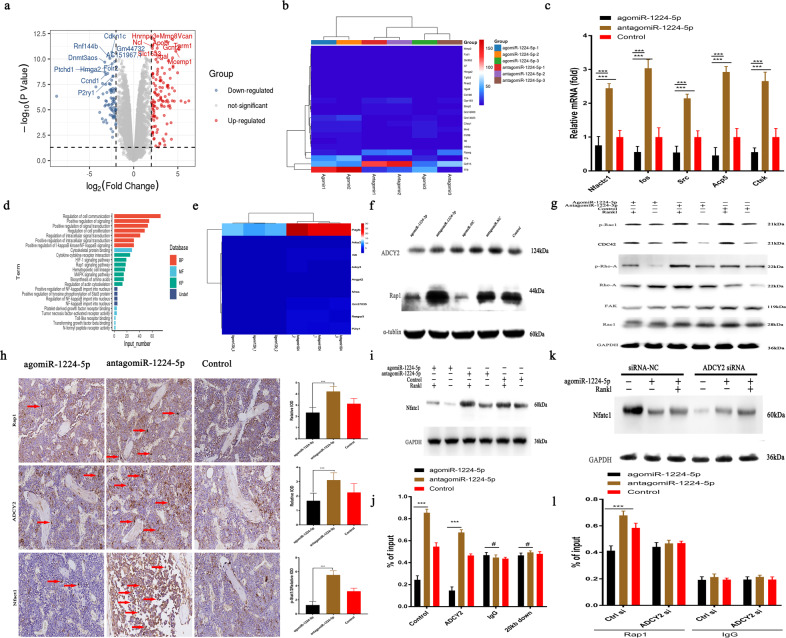
MiRNA-1224-5p inactivates the ADCY2-dependent Rap1 signaling pathway. **a** Gene expression levels of BMMs using agomiRNA-1224 and antagomiRNA-1224 24 h after transfection. The expression of 460 genes was upregulated (red), and the expression of 670 genes was downregulated (blue). Each group was treated with 30 ng/ml M-CSF. **b** Heatmap showing genes related to osteoclasts. **c** RT-PCR quantitatively confirmed that the expression of key genes in osteoclasts was upregulated. **d** KEGG pathway analysis predicts that the function of the Rap1 signaling pathway will change. **e** Heatmap shows genes related to the Rap1 pathway. **f** Western blot analysis showed that 6 h after treatment with agomiRNA-1224 in BMMs, the level of Rap1 decreased significantly. **g** Western blotting confirmed that treatment of BMMs with agomiRNA-1224 decreased the RANKL-induced protein expression of CDC42, p-Rac1, p-Rho-A, FAK, and Rac1 for 24 h. **h** Representative immunohistochemical staining showing that agomiRNA-1224 injection decreased ADCY2 and Rap1 levels in vivo. Femurs were collected within 24 h of miRNA after injection. Scale bar, 50 µm. **i** Western blotting confirmed that treatment of BMMs with agomiRNA-1224 decreased the expression of NFATC1 protein induced by RANKL for 48 h. **j** ChIP experiment results show that agomiRNA-1224 induces ADCY2 to occupy the Nfatc1-binding region together. **k** Western blot analysis of Nfatc1. The removal of ADCY2 weakened the expression of Nfatc1 induced by agomiRNA-1224. **l** ChIP experiment results show that removing ADCY2 abolishes the binding region of Rap1 and Nfatc1 triggered by agomiRNA-1224. The results are presented as the mean ± standard deviation, *N* = 3; ^#^*P* ≥ 0.05; *P < 0.05, **P < 0.01; ***P < 0.001 by *t* test.

The molecular mechanisms of the observed results were analyzed based on gene-sequencing results (detailed differential genes are presented in Supplementary material i). Compared with antagomiR-1224-5p treatment, agomiR-1224-5p treatment significantly downregulated the expression of 2632 genes, including NFACT1, a transcription factor critical for osteoclast differentiation, in addition to other target genes, such as C-FOS, SRC, ACP5, and CTSK (Fig. 5a, c). KEGG pathway analysis clearly showed that agomiRNA-1224-5p was associated with the downregulated expression of genes related to the Rap1-signaling pathway (Fig. 5d, e). We also observed that agomiR-1224-5p treatment inactivated ADCY2 and Rap1 in mouse BMMs in vitro (Fig. 5f, g). In addition, agomiR-1224-5p treatment decreased Rac1 and Rho-A expression and FAK expression induced by RANKL (Fig. 5g). Immunohistochemical staining of bone sections confirmed that following agomiR-1224-5p treatment, the number of ADCY2-, Rap1-, and NFATC1-positive cells decreased (Fig. 5h). We also observed that NFACT1 expression was downregulated after agomiR-1224-5p treatment compared with the control and antagomiRNA-1224 treatments (Fig. 5i). Chromatin immunoprecipitation (ChIP) demonstrated that, compared with the control and antagomiR-1224-5p treatment, treatment with agomiR-1224-5p significantly inhibited the recruitment of both ADCY2 and Rap1 to the binding region of Nfatc1 compared with RANKL treatment alone (Fig. 5j). We additionally investigated whether this phenomenon was dependent on ADCY2 using siRNA interference. Knockout of ADCY2 recovered the Nfatc1 expression decrease induced by agomiRNA-1224-5p (Fig. 5k) and completely abolished the triggered binding of Rap1 to Nfatc1 (Fig. 5l). The evidence above suggests that agomiR-1224-5p is dependent on Rap1 and ADCY2 to decrease the transcription of Nfact1 for inhibition of osteoclast generation.

### AgomiR-1224-5p accelerates the regeneration of bone defects and ameliorates age-related osteoporosis

**Fig. 6:**
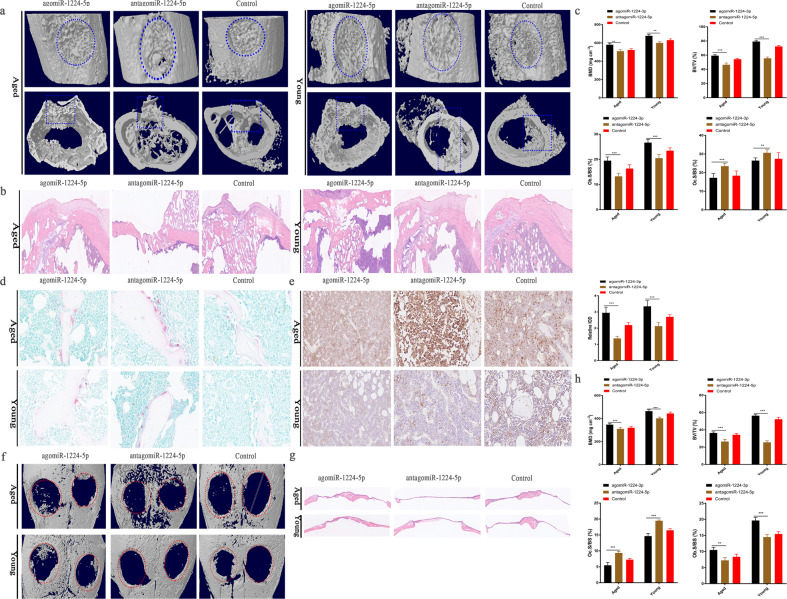
AgomiR-1224-5p accelerates the regeneration of bone defects. **a** Micro-CT reconstruction of representative femoral defects. **b** H&E-stained section of femoral defects. **c** Bone density and histomorphological analysis of new bone in the femoral cortical space. **d** Representative TRAP staining of new bone in the femoral defect. Scale bar, 50 µm. **e** Quantitative analysis of representative pictures of ADCY2 tissue immunohistochemical staining. **f** MicroCT reconstruction of skull defect. The round red dotted frame represents the size of the original bone defect model. Scale bar, 1 mm. **g** H&E-stained sections of skull defects. Scale bar, 200 µm. **h** Bone density and histomorphological analysis of new bone in the femoral cortical space. The results are presented as the mean ± standard deviation, *N* = 5; ^#^*P* ≥ 0.05; *P < 0.05, **P < 0.01; ***P < 0.001 by analysis of variance (ANOVA) with Tukey’s post-hoc test. Scale bar, 500 µm.

The role of miR-1224-5p in bone reconstruction was additionally investigated in a femoral cortical bone defect model, in which holes were created using an electric drill. Both young and elderly mice were injected with miR-1224-5p (15 μg/10 g body weight) 2 weeks prior to surgery and then scanned by μCT and analyzed by histomorphology two weeks after surgery. The results indicated that the defects in the agomiR-1224-5p group were almost entirely filled but were only partially filled in the antagomiR-1224 and PBS groups (Fig. 6a, f). The volume of mineralized callus (BV/TV) and bone density (BMD) of samples in the agomiR-1224-5p group were significantly greater than those in the control and antagomiR-1224-5p groups (Fig. 6c). The number of osteoclasts per unit of surface area in the agomiR-1224-5p group (Oc.S/BS) decreased significantly, and the number of osteoblasts (Ob.S/BS) increased significantly (Fig. 6c, d). Immunohistochemical staining demonstrated that the agomiRNA-1224-5p group had in fewer concentrated ADCY2 signals (Fig. 6e).

We then explored whether miR-1224-5p also had a similar effect on bone reconstruction in a skull model of critical bone defects. Two 1.0 mm critical bone defects were created symmetrically along the sagittal suture of the skulls of C57BL/6J mice, and miR-1224-5p was administered as described above. Only a small quantity of new bone formed in the antagomiR-1224-5p treatment group compared with that of the control and agomiR-1224-5p groups (Fig. 6f, g). Furthermore, the BV/TV and BMD values of the agomiR-1224-5p group were significantly higher than those of the control and antagomiR-1224-5p groups (Fig. 6h).

An imbalance in the formation and resorption of bone in postmenopausal women and obese patients is a pivotal cause of osteoporosis in elderly women^29^. Thus, we aimed to elucidate whether overexpression of miRNA-1224-5p could prevent bone loss caused by estrogen deficiency and intrinsic aging. First, we administered agomiR-1224-5p by continuous intraperitoneal injection (15 μg/10 g body weight, once every 3 days) in the OVX mouse model. After 6 weeks, the distal femoral metaphyses were analyzed by μCT scanning. In comparison, during the observation period, the bone loss of the mice in the agomiR-1224-5p treatment group was significantly lower (Supplementary Fig. 2a, d). More importantly, in the ovariectomized group, the number of osteoclasts (N.Oc/B.Pm) in the agomiR-1224-5p treatment group was significantly lower than that in the antagomiR-1224-5p treatment group (Supplementary Fig. 2c, d). In the sham surgery group, no significant difference was observed after treatment with antagomiR-1224-5p or controls (Supplementary Fig. 2a, d). Similarly, analysis of serum bone turnover markers demonstrated that the bone resorption marker CTX was significantly lower in the agomiR-1224-5p group (Supplementary Fig. 2e, f).

Next, we investigated the possibility of silencing miR-1224-5p for the treatment of senile osteoporosis. Eighteen-month-old mice were treated by tail vein injection with miR-1224-5p (15 μg/10 g body weight, twice per week, for 4 consecutive weeks). Analysis of the vertebral bodies in the fourth lumbar vertebra demonstrated that the volume of trabecular bone (BV/TV) in the agomiR-1224-5p treatment group was significantly higher (Supplementary Fig. 2g, i). The trabecular bone number (Tb.N) and thickness (Tb.Th) values also increased significantly, while the trabecular bone separation (Tb.Sp) value decreased (Supplementary Fig. 2j). In summary, the results demonstrate that increased miR-1224-5p expression may represent a potential target for postmenopausal and senile osteoporosis and the treatment of nonunion bone fractures.

## Discussion

The present study identified miR-1224-5p as an important differentiation-inhibiting factor in osteoclasts in both normal aging physiological conditions and following a fracture. The results indicate that overexpression of miR-1224-5p in osteoclasts may reduce bone resorption and even increase bone formation by modifying osteoclast function and osteoblast differentiation.

Many miRNA molecules are important regulators of bone resorption-related gene expression at the post-transcriptional level^30^. However, few miRNAs that act on bone homeostasis in the context of fractures have been identified^31,32^. In the present study, we found that the expression levels of miR-1224-5p increased significantly after fracture and during fracture healing and were lower in osteoporotic patients. This clinical result indicated that miR-1224-5p is involved in bone formation and resorption during fracture healing and osteoporosis. Therefore, we explored whether miR-1224-5p mediates bone healing and osteoporosis development in bone defect and fracture and OVX mouse models. We found that bone mass was significantly higher in the agomiR-1224-5p treatment group. In addition, the rate of bone formation and the callus volume were significantly higher in the fracture + agomiR-1224-5p group. This finding can be explained by the positive synergistic effects of bone reconstruction and bone formation in these models. In senile bone formation in ovariectomized mice, this phenomenon may be mainly due to the decreased bone resorption by inhibition of osteoclastogenesis, while in the fracture models, inhibition of osteoclastogenesis and the promotion of osteoblastogenesis may promote both bone formation and fracture healing.

It has been previously reported that miR-1224 inhibits the proliferation of fibroblasts and promotes their apoptosis through the TGFβ1/Smad3-signaling pathway, which is highly expressed in exosomes in blood and is able to promote osteoclast formation via the Hippo-signaling pathway. This molecule can also interact with circular RNA-Filip1L, thereby targeting Ubr5 and regulating chronic inflammatory pain^11,13,15^. This finding indicates that miR-1224-5p is involved in the neuroregulation of peripheral fibrous tissue and the regulation of osteoclasts. However, there have been no previous reports that miR-1224-5p can activate the Rap1-signaling pathway by targeting ADCY2, preventing osteoclastogenesis and promoting osteoblast differentiation. In the present study, a new mechanism was proposed in which miR-1224-5p regulates osteoclasts by targeting ADCY2, inactivating the Rap1-signaling pathway, and decreasing the protein expression of ADCY2, Rac1, Rho-A, Rac1, and FAK. Previous studies have shown that the normal gene expression of FAK, Rac1/2/3, Rac1/CDC42, and Rho-A is key to the formation of osteoclast pseudopodia, cell adhesion, and motor function, with the Rap1-signaling pathway involved in the regulation^33,34^. In the past few decades, osteoclasts have been considered end-stage cells^35^. After fulfilling their absorption function, these cells undergo apoptosis and are then phagocytosed and cleared by lysosomes. We found that overexpression of miR-1224-5p significantly reduced the expression of FAK, Rac1/2/3, Rac1/CDC42, and Rho-A, which are related to the cytoskeletal structure and important for the morphology and function of osteoclasts. Considering the recently discovered osteomorphs^36^, we suspect that miR-1224-5p may simultaneously affect the recycling of osteoclasts, but we have not been able to verify this supposition. ADCY2 inactivation effectively decreases osteoclast formation and function by inhibiting osteoclast-specific gene expression. The data in the present study confirm that miR-1224-5p downregulated ADCY2 protein expression. In addition, the in vivo and in vitro evidence strongly suggests that ADCY2 may be a functional target protein of miR-1224-5p and may mediate the regulation of bone resorption.

Notably, this study has a number of limitations. First, although we found that miR-1224-5p affects the bone resorption capability of osteoclasts, it is difficult to effectively provide targeted miR-1224-5p overexpression in osteoclasts in osteoporosis and fractured local bone. Nevertheless, our research first showed that miRNA promotes bone resorption in osteoclasts both in vivo and in vitro, and in diseased conditions, specific overexpression of miR-1224-5p can harmonize osteoclast and osteoblast differentiation that promotes bone formation. We view this study as a novel step toward developing a potential therapeutic target for osteoporosis and fracture nonunion.

## Supplementary information

supplementary materials

## Data Availability

The data used to support the findings of this study are available from the corresponding author upon request.

## References

[1] Gosch M, Kammerlander C, Neuerburg C (2019). Osteoporosis-epidemiology and quality of care. Z. Gerontol. Geriatr..

[2] Tsou AY (2020). Medical care of adults with Down syndrome: a clinical guideline. JAMA.

[3] Lorentzon M (2019). Treating osteoporosis to prevent fractures: current concepts and future developments. J. Intern. Med..

[4] Odén A, McCloskey EV, Johansson H, Kanis JA (2013). Assessing the impact of osteoporosis on the burden of hip fractures. Calcif. Tissue Int..

[5] Kanis JA, Cooper C, Rizzoli R, Reginster JY (2019). European guidance for the diagnosis and management of osteoporosis in postmenopausal women. Osteoporos. Int..

[6] Reid IR (2020). A broader strategy for osteoporosis interventions. Nat. Rev. Endocrinol..

[7] Khosla S, Hofbauer LC (2017). Osteoporosis treatment: recent developments and ongoing challenges. Lancet Diabetes Endocrinol..

[8] Zhu S (2018). Coupling factors and exosomal packaging microRNAs involved in the regulation of bone remodelling. Biol. Rev. Camb. Philos..

[9] Kagiya T, Taira M (2013). Expression of microRNAs in the extracellular microvesicles of murine osteoclasts. J. Oral Tissue Eng..

[10] Lin C (2019). Circulating miR-338 cluster activities on osteoblast differentiation: potential diagnostic and therapeutic targets for postmenopausal osteoporosis. Theranostics.

[11] Pan Z (2019). MicroRNA-1224 splicing circularRNA-Filip1l in an Ago2-dependent manner regulates chronic inflammatory pain via targeting Ubr5. J. Neurosci. Res..

[12] Lyu L (2019). Integrated interaction network of microRNA target genes in keloid scarring. Mol. Diagn. Ther..

[13] Niu Y (2011). Lipopolysaccharide-induced miR-1224 negatively regulates tumour necrosis factor-α gene expression by modulating Sp1. Immunology.

[14] Singleton Q (2019). Bone marrow derived extracellular vesicles activate osteoclast differentiation in traumatic brain injury induced bone loss. Cells.

[15] Li B (2019). The role and mechanism of miRNA-1224 in the *Polygonatum sibiricum* polysaccharide regulation of bone marrow-derived macrophages to osteoclast differentiation. Rejuvenation Res..

[16] Weischenfeldt J, Porse B (2008). Bone marrow-derived macrophages (BMM): isolation and applications. Csh. Protoc..

[17] Sreejit P, Dilip KB, Verma RS (2012). Generation of mesenchymal stem cell lines from murine bone marrow. Cell Tissue Res..

[18] Tropel P (2004). Isolation and characterisation of mesenchymal stem cells from adult mouse bone marrow. Exp. Cell Res..

[19] Liu B (2019). A protocol for isolation and identification and comparative characterization of primary osteoblasts from mouse and rat calvaria. Cell Tissue Bank.

[20] Liu W (2016). GDF11 decreases bone mass by stimulating osteoclastogenesis and inhibiting osteoblast differentiation. Nat. Commun..

[21] Y X (2019). miRNA-26a-5p accelerates healing via downregulation of PTEN in fracture patients with traumatic brain injury. Mol. Ther.-Nucleic Acids.

[22] Liu W (2016). Effect of resorbable collagen plug on bone regeneration in rat critical-size defect model. Implant Dent..

[23] Kim J (2012). Fiber-reinforced calcium phosphate cement formulations for cranioplasty applications: a 52-week duration preclinical rabbit calvaria study. J. Biomed. Mater. Res. B..

[24] Spicer PP (2012). Evaluation of bone regeneration using the rat critical size calvarial defect. Nat. Protoc..

[25] Hu L (2021). miRNA-92a-3p regulates osteoblast differentiation in patients with concomitant limb fractures and TBI via IBSP/PI3K-AKT inhibition. Mol. Ther.-Nucleic Acids.

[26] Souza VR (2019). Description of ovariectomy protocol in mice. Methods Mol. Biol..

[27] Kim JM, Lin C, Stavre Z, Greenblatt MB, Shim JH (2020). Osteoblast–osteoclast communication and bone homeostasis. Cells.

[28] Onishi M, Fujita Y, Yoshikawa H, Yamashita T (2013). Inhibition of Rac1 promotes BMP-2-induced osteoblastic differentiation. Cell Death Dis..

[29] Eastell R (2016). Postmenopausal osteoporosis. Nat. Rev. Dis. Prime.

[30] Dexheimer PJ, Cochella L (2020). MicroRNAs: from mechanism to organism. Front. Cell Dev. Biol..

[31] Komatsu DE, Duque E, Hadjiargyrou M (2021). MicroRNAs and fracture healing: pre-clinical studies. Bone.

[32] Nugent M (2017). MicroRNAs and fracture healing. Calcif. Tissue Int..

[33] Mediero A, Perez-Aso M, Cronstein BN (2014). Activation of EPAC1/2 is essential for osteoclast formation by modulating NFκB nuclear translocation and actin cytoskeleton rearrangements. FASEB J..

[34] Zou W (2013). Talin1 and Rap1 are critical for osteoclast function. Mol. Cell. Biol..

[35] Bonucci E (1981). New knowledge on the origin, function and fate of osteoclasts. Clin. Orthop. Relat. Res..

[36] McDonald MM (2021). Osteoclasts recycle via osteomorphs during RANKL-stimulated bone resorption. Cell.

