# Supplementary material for: MiR-1224-5p modulates osteogenesis by coordinating osteoblast/osteoclast differentiation via the Rap1 signaling target ADCY2
Source: Exp Mol Med. 2022 Jul 13;54(7):961–72. doi: 10.1038/s12276-022-00799-9 (PMC9355958; doi:10.1038/s12276-022-00799-9)
Supplement: Supplementary file 1 — supplementary materials [file 12276_2022_799_MOESM1_ESM.pdf]

Supplementary materials text summary

a. Supplementary Fig. 1

b. Supplementary Fig. 2

c. Supplementary Fig. 3

d. Supplementary Fig. 4

e. Illustrate model

f. Supplementary figure legends

g. Supplementary words

h. Supplementary table. 1

i. Sequencing different genes

j. Sequencing result-sig_UP_miRNAs

a. Supplementary Fig. 1

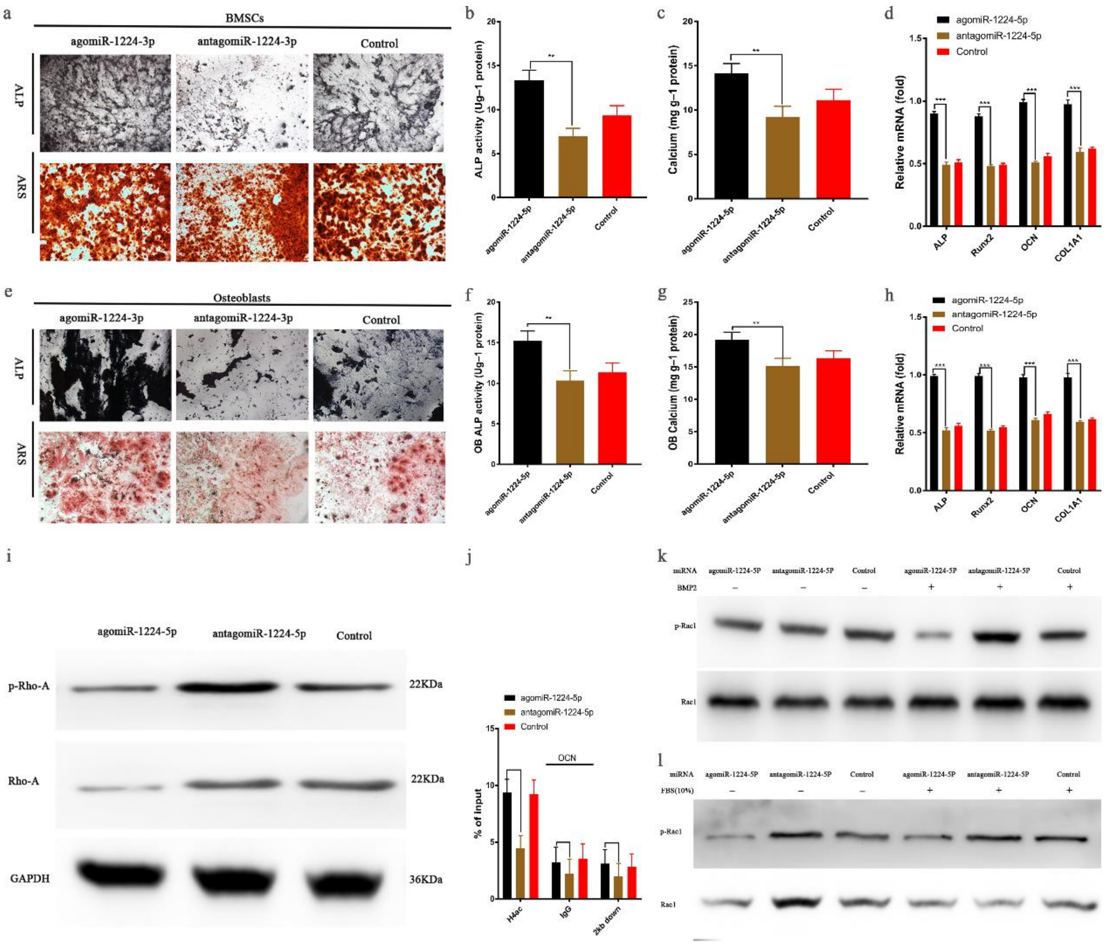

b. Supplementary Fig. 2

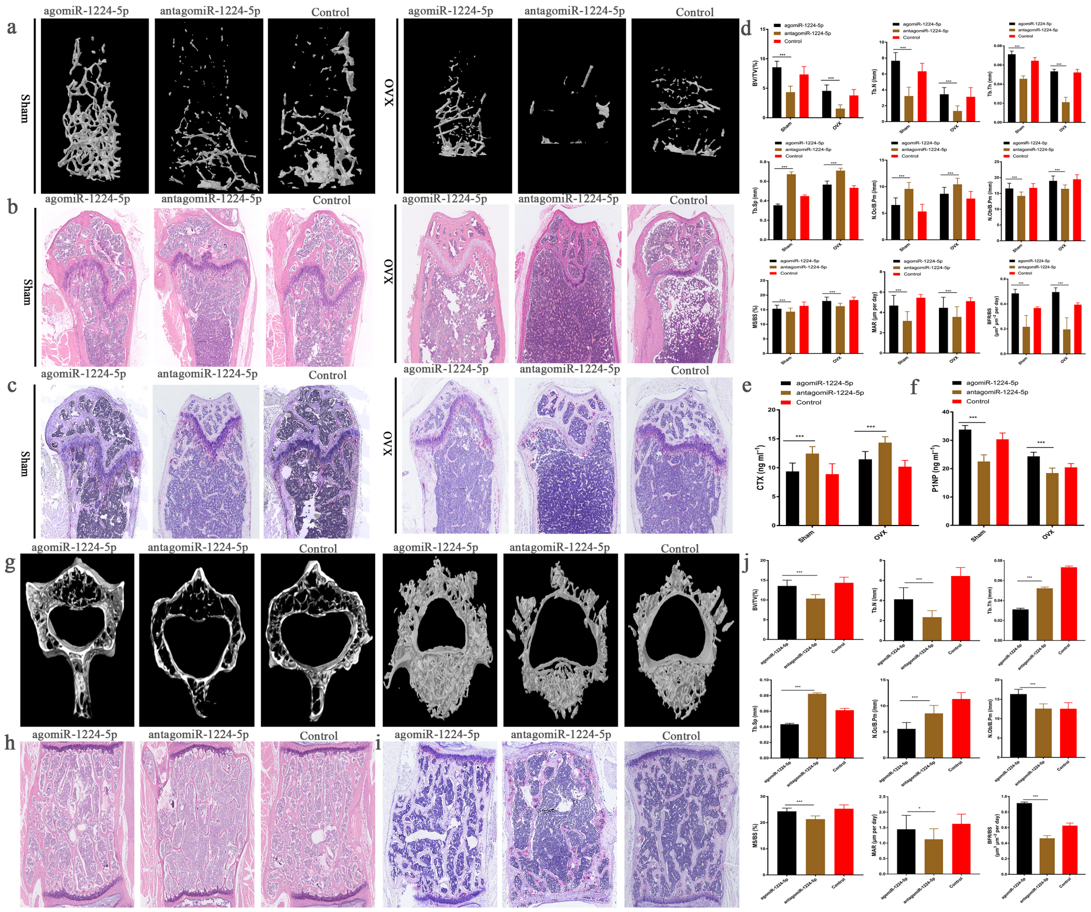

c. Supplementary Fig. 3

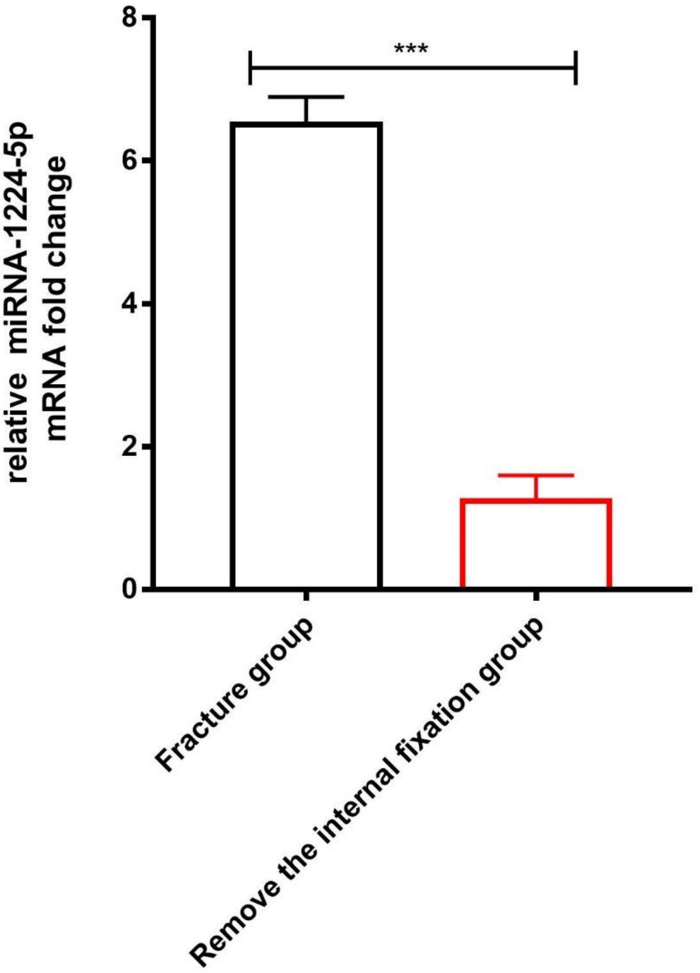

d. Supplementary Fig.4

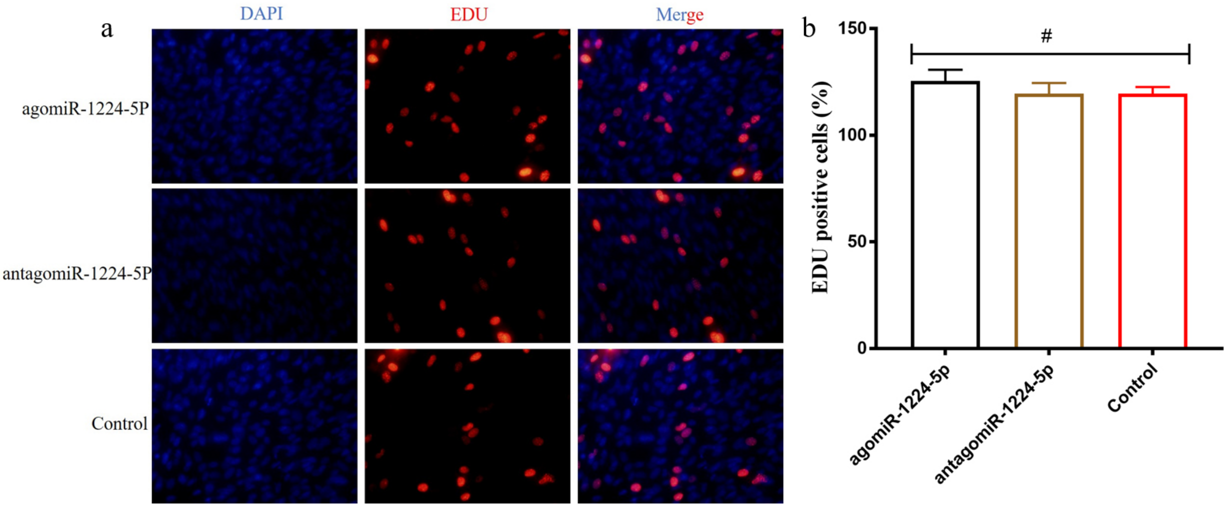

e. Illustrate model

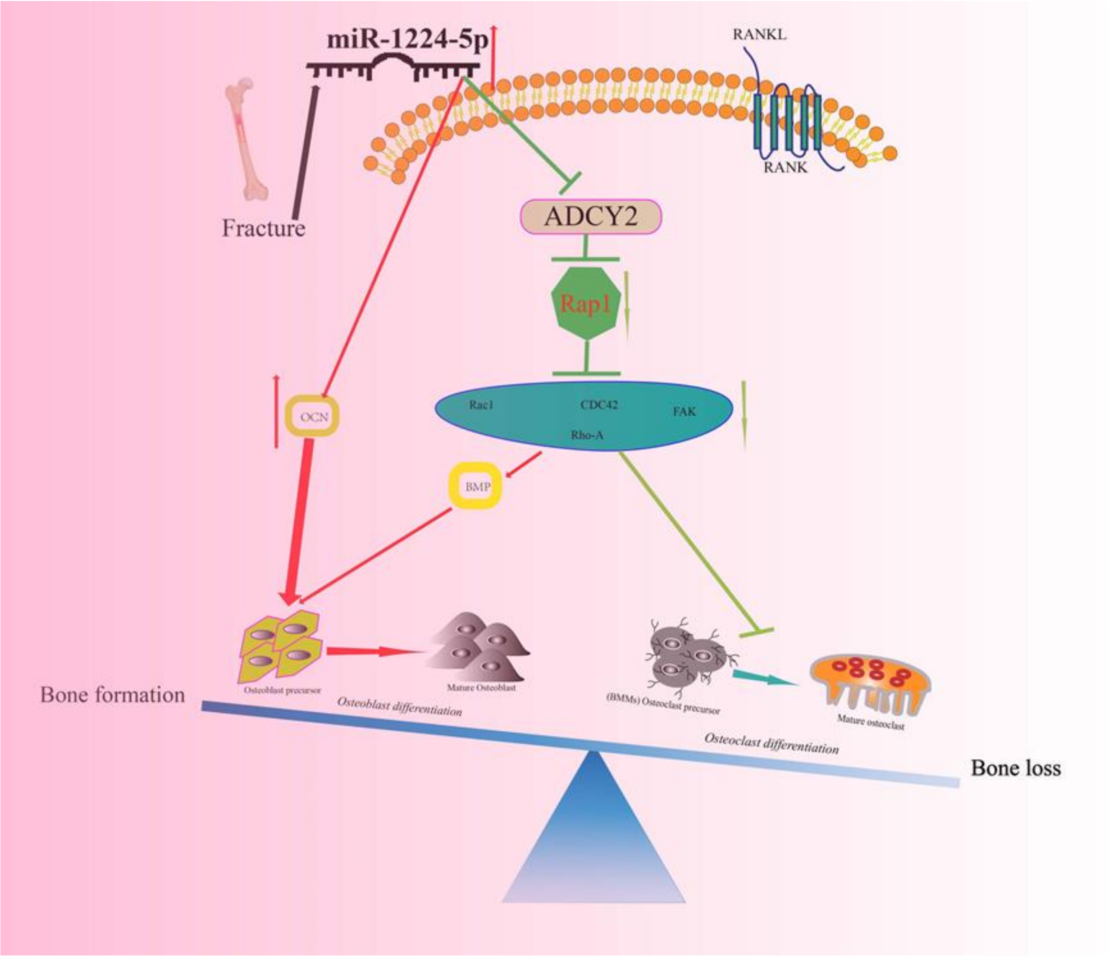

f. Supplementary figure legends

Supplementary Fig1. AgomiR-1224-5p stimulates osteoblast differentiation.

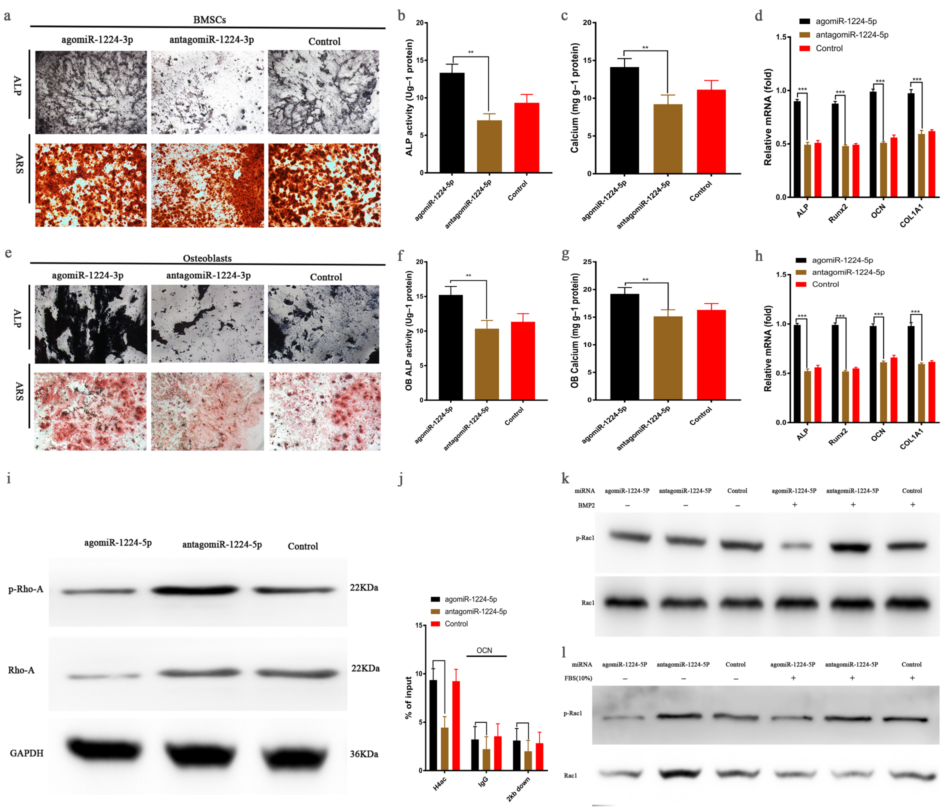

a. Representative ALP staining and Alizarin Red S staining (Alizarin Red S). b-c. quantitative analysis of ALP activity and calcium mineralization of BMSCs. d. RT-PCR showed that 24h after agomiRNA-1224 treated BMSCs, the mRNA expression levels of osteogenic genes (ALP, Runx2, Ocn, Osx) decreased. e. Representative ALP staining and Alizarin Red S. f-g. Quantitative analysis of ALP activity and calcium mineralization of BMSCs. h. RT-PCR showed that 24h after agomiRNA-1224 treated skull osteoblast precursor cells, the mRNA expression levels of osteogenic genes (ALP, Runx2, Ocn, Osx) decreased. i. Western-Blot confirmed that the treatment of osteoblasts with agomiRNA-1224 decreased the phosphorylation level of Rho-A for 48 hours. j. Chip experiment results show that agomiRNA1224 reduces the protein abundance of acetylated histone H4 (H4ac) in Ocn promoter. k-l. Western-Blot analysis of p-Rac1. agomiRNA-1224 attenuated the phosphorylation of Rac1 induced by BMP2 or fetal bovine serum, $^{\#}P \geq 0.05$; $^{*}P<0.05$, $^{**}P<0.01$; $^{***}P<0.001$ by t test, N=3.

Supplementary Fig.2. AntagomiR-1224-5p impairs OVX-induced bone loss.

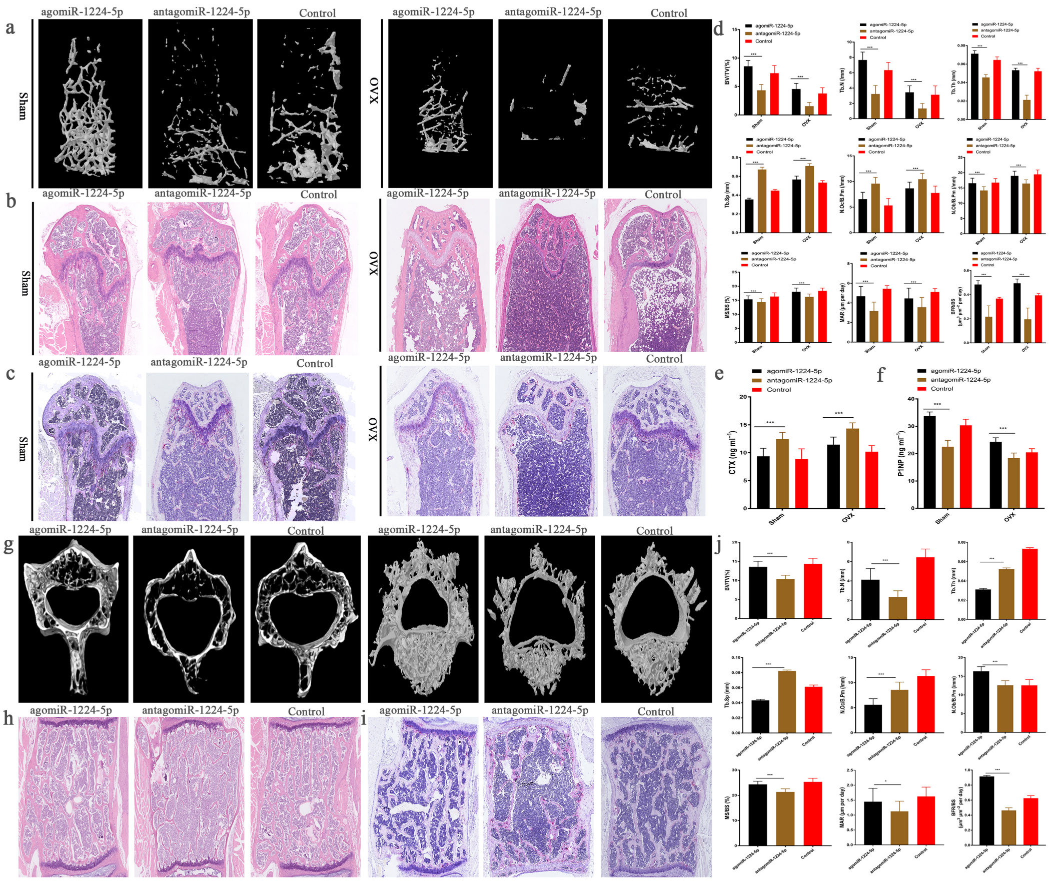

a. MicroCT reconstruction of the trabecular bone of the distal femur. b. HE stained sections of the distal femur without decalcification. c. TRAP staining of distal femur. Scale bar, 500um. d. Histomorphological analysis of the metaphyseal region of the distal femur. e-f. Serum CTX and P1NP expression levels. g. MicroCT reconstructs the trabecular bone of the lumbar 4 vertebral body, the red circle dotted line represents the region of interest (ROI). Scale bar, 1mm. h. HE stained lumbar 4 vertebral sections without decalcification. i. TRAP staining of lumbar 4 vertebral sections. j. Histomorphological analysis of lumbar 4 vertebrae. The results are presented in the mean ± standard deviation, $^{\#}p \geq 0.05$; $^{*}P<0.05$, $^{**}P<0.01$; $^{***}p<0.001$ by t test, N=3.

Supplementary Fig.3. Relative miRNA-1224-5p mRNA fold change between fracture patients and normal (remove the internal fixation) patients within 1 week after fracture.

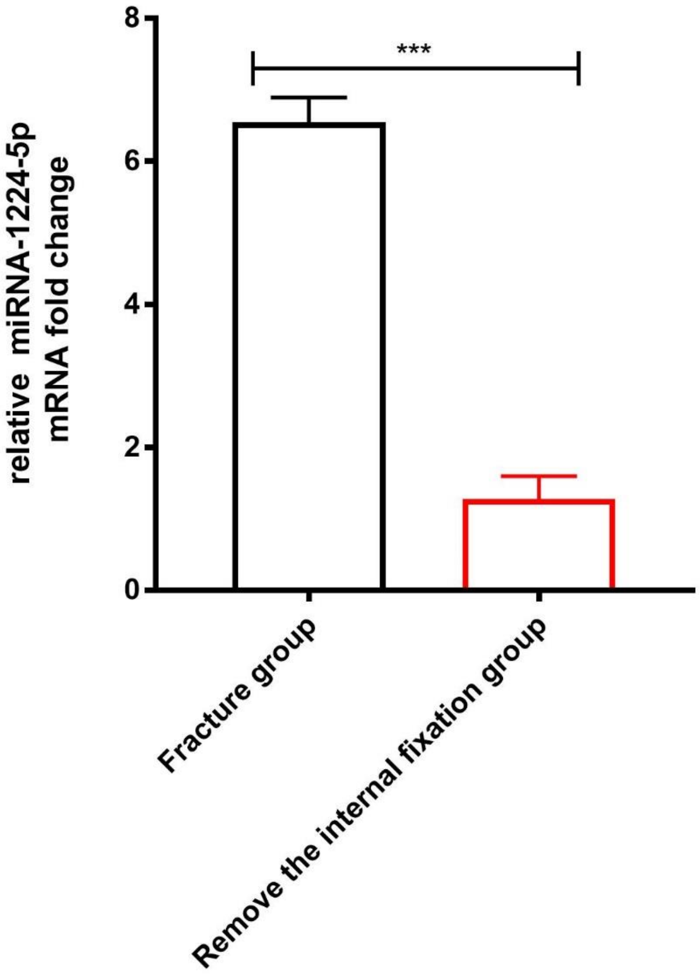

To further confirm the expression of miRNA-1224-5p in fracture patients and normal (Remove the internal fixation) patients within 1 week after fracture, we again collected the peripheral blood of 10 normal patients and fracture patients of the same age and same AO classification for RT-PCR, the results confirmed that miRNA-1224-5p expression in the fracture group was significantly up-regulated and statistically significant. (N = 10, ***P < 0.01)

Supplementary Fig.4. The effect of miRNA-1224-5p on BMSC proliferation

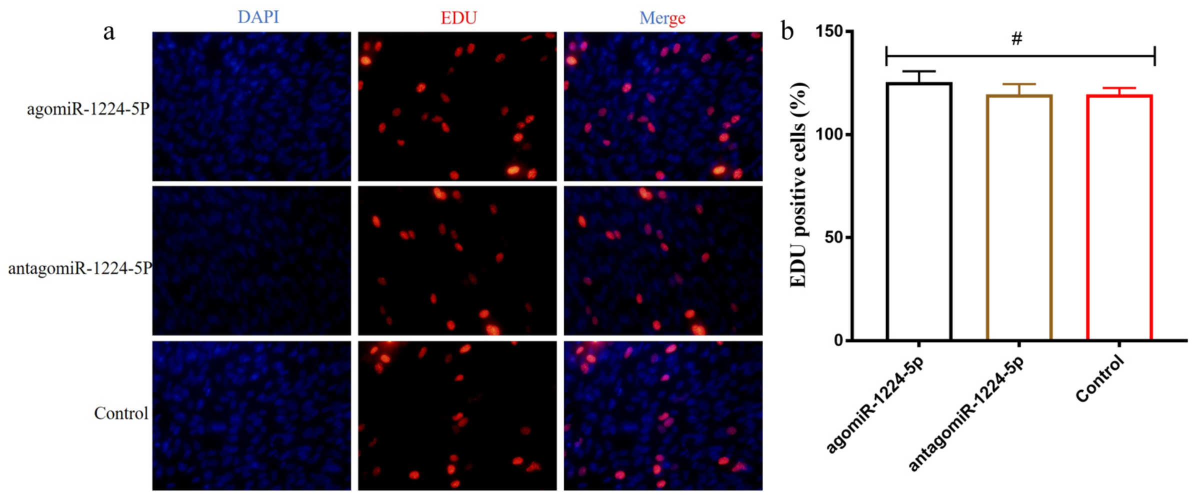

AntagomiRNA-1224-5p was used to silence endogenous mirNA-1224-5P in BMSCs, and the EDU staining proliferation results showed no statistical difference compared with overexpression and blank control group (bar 40X, #P>0.05, N=3).

g. Supplementary words

Abbreviation and Credit author statement

ADCY2, adenylate cyclase 2; FAK, nuclear focal adhesion kinase; NFATC1, nuclear factor of activated T-cells cytoplasmic 1; CTX, C-terminal telopeptide ofβ-I collagen; OVX, ovariectomy; BMD, bone mineral density; RANKL, nuclear factor-kappa B ligand; BMMs, bone marrow-derived macrophages; BMSCs, bone marrow mesenchymal stem cells; TRAP, tartrate-resistant acid phosphatase; SRC, Proto-oncogene tyrosine-protein kinase ; CTSK, cathepsin K; μCT, micro-computed tomography; H&E, hematoxylin and eosin

Credit author statement

L.H., B.M., and G.L. conceived and designed the experiments. X.D., H.X., Z.L., B.M., G.L., C.Y., L.C., Y.H., R.Y., C.Y. and W.Z. performed the experiments. L.H., H.X., Y.S., P.C., K.Z., and G.L. analyzed the data and prepared the figures. X.X., J.L., H.X., T.W., and Z.L. contributed reagents, materials, and analysis tools. K.Z., P.C., D.X, and G.L. performed statistical analyses. L.H., B.M. and A.C.P. wrote the manuscript.

h. Supplementary Table 1. Clinical information of the patients included in the study

Patients	Gender (Female/Male)	Age (year)	Fracture or Remove the internal fixation	Fracture position	Fracture classification (AO classification)	Time form fracture /surgery to blood sample collection (Day)
1	Female	35	Fracture	Right femur	31-A1	6
2	Female	47	Fracture	Left femur	31-A1	6
3	Female	46	Fracture	Right femur	31-A1	7
4	Male	37	Fracture	Left femur	31-A1	7
5	Male	40	Fracture	Left femur	31-A2	8
6	Female	28	Fracture	Right femur	31-A2	6
7	Male	36	Fracture	Left femur	31-A2	6
8	Male	35	Fracture	Right femur	31-A2	6
9	Male	33	Fracture	Left femur	31-A1	7
10	Female	40	Fracture	Left femur	31-A1	7
11	Male	37	Remove the internal fixation	Right femur	31-A2	7
12	Female	37	Remove the internal fixation	Left femur	31-A2	8
13	Female	38	Remove the internal fixation	Right femur	31-A1	6
14	Male	41	Remove	Left	31-A2	6

			the internal fixation	femur		
15	Female	31	Remove the internal fixation	Left femur	31-A2	6
16	Male	34	Remove the internal fixation	Right femur	31-A1	7
17	Male	36	Remove the internal fixation	Left femur	31-A2	7
18	Male	35	Remove the internal fixation	Right femur	31-A1	7
19	Female	38	Remove the internal fixation	Left femur	31-A1	7
20	Male	33	Remove the internal fixation	Left femur	31-A2	8

i. Sequencing different genes

Geneid	logFC	PValue	FDR
Mmp8	2.734731	5.77E-13	5.27E-09
Hnrnpa3	2.288443	1.16E-12	5.27E-09
Vcan	4.427951	1.21E-12	5.27E-09
Gcnt2	3.748988	1.88E-12	6.14E-09
Apobr	2.181613	4.31E-12	1.13E-08
Ncl	2.106845	5.63E-12	1.23E-08
Hk2	1.982888	6.97E-12	1.3E-08
Itgal	2.669073	8.68E-12	1.32E-08
Slc16a3	2.124069	9.24E-12	1.32E-08
Tarm1	5.133527	1.15E-11	1.36E-08
Mcemp1	4.765556	1.71E-11	1.87E-08
Atrnl1	2.464068	1.95E-11	1.96E-08
Pfkfb3	1.339292	7.02E-11	5.73E-08
F5	4.404913	7.69E-11	5.91E-08
Rrbp1	1.380554	9.4E-11	6.14E-08
Tgfbi	1.480832	1.01E-10	6.28E-08
Hnrnpa2b1	1.514933	1.1E-10	6.34E-08
Fam98b	2.511746	1.12E-10	6.34E-08
Dmxl2	1.45761	1.28E-10	6.94E-08
Trim41	1.712273	1.36E-10	7E-08
Il6ra	1.75692	1.39E-10	7E-08
Dhx9	3.91749	1.54E-10	7.28E-08
Bst1	1.756109	1.7E-10	7.41E-08
Aoah	1.427383	1.83E-10	7.47E-08
Gm24574	2.711386	2.03E-10	7.68E-08
Pilra	1.936575	2.05E-10	7.68E-08
Gys1	1.417334	2.52E-10	9.13E-08
Gm17509	1.515924	3.79E-10	1.24E-07
N4bp1	1.208114	4E-10	1.26E-07
Gda	1.181325	4.06E-10	1.26E-07
Pgk1	1.157538	4.5E-10	1.37E-07
Orm1	4.45683	4.92E-10	1.46E-07
Fgr	1.202742	5.04E-10	1.46E-07
Iqgap2	1.295163	5.87E-10	1.62E-07
Slc15a3	1.356743	6.28E-10	1.62E-07
Prdx5	1.113797	6.51E-10	1.62E-07
Hnrnpul2	1.598323	6.64E-10	1.62E-07
Nos2	4.456904	6.67E-10	1.62E-07
F10	3.220711	7.31E-10	1.73E-07
Sgms2	2.730729	9.89E-10	2.3E-07

Emilin2	1.548358	1E-09	2.3E-07
Taf15	4.08028	1.04E-09	2.34E-07
Pkm	0.977839	1.21E-09	2.63E-07
Anp32a	1.154748	1.48E-09	3.06E-07
Gpr141	1.339332	1.69E-09	3.39E-07
Gm6254	2.705723	1.75E-09	3.46E-07
Hif1a	1.464845	1.8E-09	3.5E-07
9430076C15Rik	1.018876	1.99E-09	3.77E-07
Hexim1	1.252768	2.07E-09	3.82E-07
Cdk11b	0.980608	2.25E-09	4.08E-07
Fpr1	4.97194	2.28E-09	4.09E-07
Peli2	1.325514	2.34E-09	4.12E-07
Pygl	1.062218	2.59E-09	4.33E-07
Fgd3	2.160292	2.6E-09	4.33E-07
Safb2	1.573013	2.63E-09	4.33E-07
A730081D07Rik	3.488079	2.65E-09	4.33E-07
Gapdh	0.950552	2.66E-09	4.33E-07
Gpi1	0.985145	2.8E-09	4.42E-07
Sirpb1c	1.371518	2.81E-09	4.42E-07
Slc31a1	1.014653	2.93E-09	4.51E-07
Glipr2	2.180522	2.94E-09	4.51E-07
Aldoa	0.927152	3E-09	4.51E-07
Eno1	0.935829	3.1E-09	4.6E-07
Gm26762	1.138327	3.21E-09	4.68E-07
Ptgfrn	2.677603	3.26E-09	4.68E-07
Sirpb1b	1.012196	3.41E-09	4.68E-07
Hnrnph3	2.092017	3.42E-09	4.68E-07
Eif3a	1.014629	3.44E-09	4.68E-07
Cenpb	0.948641	3.51E-09	4.73E-07
Map4k4	1.1164	3.59E-09	4.79E-07
Emb	1.485154	4.38E-09	5.67E-07
Fam57b	0.979852	4.64E-09	5.86E-07
Tnks1bp1	1.353608	4.67E-09	5.86E-07
Il13ra1	1.692365	5.05E-09	6.14E-07
Sart1	0.954231	5.08E-09	6.14E-07
Gas8	1.009385	5.19E-09	6.16E-07
Pdk1	1.059349	5.62E-09	6.55E-07
Bnip3	1.37717	6.01E-09	6.82E-07
Fbxl20	0.95126	6.03E-09	6.82E-07
C3	1.157091	6.12E-09	6.82E-07
Ccdc88b	1.040192	6.24E-09	6.82E-07
Ero1l	1.131613	6.26E-09	6.82E-07
Myh9	0.97471	6.27E-09	6.82E-07

Pbxip1	1.545969	6.36E-09	6.86E-07
Slpi	3.004019	6.56E-09	6.95E-07
Pfkl	2.212645	6.63E-09	6.95E-07
Kif3a	1.227889	6.65E-09	6.95E-07
Pag1	1.154781	7.58E-09	7.77E-07
Lpcat2	1.180583	7.61E-09	7.77E-07
Gbp11	4.139602	7.88E-09	7.98E-07
Ednrb	1.815144	8.43E-09	8.47E-07
Plekhg1	1.861863	9.37E-09	9.13E-07
Tpi1	0.982453	9.46E-09	9.15E-07
Ccr1	2.53033	1.06E-08	1.01E-06
Svil	0.975662	1.1E-08	1.04E-06
Smpdl3b	3.114575	1.23E-08	1.11E-06
Cd302	1.860093	1.26E-08	1.13E-06
Mllt6	0.965744	1.29E-08	1.14E-06
Arg2	2.980171	1.33E-08	1.17E-06
Abca1	1.122304	1.33E-08	1.17E-06
Tifab	1.131418	1.38E-08	1.2E-06
Tle3	1.158955	1.46E-08	1.23E-06
Marco	3.025558	1.46E-08	1.23E-06
Ikbke	0.82564	1.49E-08	1.24E-06
Dynll2	1.133425	1.5E-08	1.24E-06
Pstpip2	1.288192	1.52E-08	1.25E-06
Fcgr2b	1.284202	1.53E-08	1.25E-06
Cers6	0.912604	1.54E-08	1.25E-06
Mtus1	1.035797	1.73E-08	1.38E-06
Ric1	0.884163	1.89E-08	1.47E-06
Rnf149	1.069529	1.9E-08	1.47E-06
Cep250	0.82522	1.99E-08	1.52E-06
Egln3	3.368402	2.17E-08	1.6E-06
Abtb2	1.936214	2.18E-08	1.6E-06
Pgm2	0.859978	2.21E-08	1.6E-06
Mr1	1.298637	2.25E-08	1.62E-06
Tfec	0.988927	2.29E-08	1.64E-06
Chsy1	1.203888	2.31E-08	1.64E-06
Fbl	0.942545	2.33E-08	1.64E-06
Ly6i	2.266988	2.51E-08	1.75E-06
Pdpn	1.411719	2.57E-08	1.78E-06
Gm15283	1.704913	2.62E-08	1.8E-06
Arhgap24	0.960248	2.65E-08	1.81E-06
Ms4a4a	1.50758	2.68E-08	1.82E-06
Dram1	1.206444	3.36E-08	2.16E-06
P4ha1	0.830517	3.57E-08	2.24E-06

H2-M2	3.897417	3.89E-08	2.42E-06
Siglece	3.816391	4.03E-08	2.48E-06
Jak2	1.129746	4.05E-08	2.48E-06
Mir5114	2.461771	4.28E-08	2.58E-06
Pla2g4a	1.185792	4.42E-08	2.63E-06
Setd7	0.841581	4.6E-08	2.68E-06
Akap13	0.996778	4.74E-08	2.73E-06
Mgst1	1.071904	4.87E-08	2.76E-06
Cox6a2	1.831674	4.96E-08	2.78E-06
Pla2g7	0.88929	5.29E-08	2.93E-06
Fpr2	3.796816	5.32E-08	2.93E-06
Ift57	1.043466	5.66E-08	3.07E-06
Sema4a	0.94421	5.81E-08	3.13E-06
Gm4735	0.965501	6.08E-08	3.23E-06
Mmp14	2.965708	6.11E-08	3.23E-06
RP23-320D23.6	0.874905	6.33E-08	3.34E-06
Adora2a	3.260758	6.62E-08	3.47E-06
1700071M16Rik	1.067959	6.77E-08	3.51E-06
Slc2a1	1.620691	6.94E-08	3.58E-06
Inhba	3.92511	7.07E-08	3.61E-06
Fpr3	4.370846	7.12E-08	3.61E-06
Add3	0.968797	7.28E-08	3.67E-06
Rnf145	1.033938	7.84E-08	3.92E-06
Cybb	1.162594	8.08E-08	4E-06
Safb	1.248677	8.43E-08	4.09E-06
Gbp7	0.986119	8.58E-08	4.14E-06
Narf	1.03746	8.59E-08	4.14E-06
Gngt2	0.963832	8.85E-08	4.25E-06
Phyhd1	1.16362	9.24E-08	4.41E-06
Slc36a1	0.822958	9.25E-08	4.41E-06
Ddhd1	1.607639	9.48E-08	4.48E-06
Mvd	1.191667	9.52E-08	4.48E-06
Scd2	2.538939	9.58E-08	4.48E-06
Mpst	1.12752	9.61E-08	4.48E-06
Gm45472	1.046777	9.64E-08	4.48E-06
Sh2b2	1.293766	9.77E-08	4.53E-06
Trim56	0.809775	9.99E-08	4.59E-06
Dock4	1.110812	1.04E-07	4.77E-06
Anp32e	0.808581	1.06E-07	4.82E-06
Arl5c	1.345962	1.08E-07	4.84E-06
Hirip3	1.380528	1.11E-07	4.94E-06
Gm44250	1.06908	1.29E-07	5.52E-06
Gm15832	0.997487	1.35E-07	5.7E-06

Cd38	4.627288	1.38E-07	5.79E-06
Lamc1	0.836129	1.39E-07	5.82E-06
Fbxl5	0.818671	1.44E-07	5.96E-06
Gm11847	3.087682	1.5E-07	6.19E-06
Bcl2a1b	1.201635	1.53E-07	6.25E-06
Hmgn5	1.471795	1.53E-07	6.26E-06
Tnfrsf14	1.056823	1.56E-07	6.33E-06
Trpm2	1.773975	1.57E-07	6.35E-06
Abca7	0.995886	1.57E-07	6.35E-06
Gm5641	2.357929	1.81E-07	7.17E-06
Tma16	1.89237	2.05E-07	7.82E-06
Sod2	2.412242	2.14E-07	8E-06
Phgdh	1.644283	2.14E-07	8E-06
Palm	1.452233	2.15E-07	8E-06
Gm45507	1.358009	2.27E-07	8.32E-06
Orai2	0.895251	2.27E-07	8.32E-06
Herc2	0.825392	2.3E-07	8.34E-06
Kctd17	1.676563	2.31E-07	8.34E-06
Zfp609	0.990188	2.33E-07	8.39E-06
Emilin1	0.90257	2.46E-07	8.74E-06
Cdc42bpg	1.422361	2.51E-07	8.89E-06
Gm6793	1.849027	2.59E-07	9.11E-06
Procr	1.768349	2.63E-07	9.18E-06
Gm9242	2.528408	2.94E-07	9.95E-06
Ak4	4.011821	2.99E-07	1E-05
Gsap	1.161695	3E-07	1E-05
Acpp	2.816605	3.17E-07	1.05E-05
F830208F22Rik	1.32754	3.58E-07	1.16E-05
Itgam	0.817247	3.91E-07	1.25E-05
Ptges	2.677896	3.96E-07	1.26E-05
0610009E02Rik	1.383032	3.96E-07	1.26E-05
Notch1	1.627889	4.28E-07	1.33E-05
Ift172	0.831212	4.3E-07	1.33E-05
Tmem268	0.893279	4.46E-07	1.37E-05
Gm16675	1.635651	4.63E-07	1.41E-05
Gm16192	3.567358	4.92E-07	1.48E-05
Arhgef37	3.062228	5.75E-07	1.66E-05
Nuak1	0.978101	5.81E-07	1.67E-05
Tbc1d9	0.895545	6E-07	1.71E-05
Pgk1-rs7	1.083084	6.18E-07	1.75E-05
Wipi1	0.9356	6.25E-07	1.77E-05
Lrba	0.885047	6.31E-07	1.77E-05
Osbpl3	1.747424	6.5E-07	1.81E-05

Nectin2	0.879606	6.52E-07	1.81E-05
Dtx3	0.98082	6.71E-07	1.84E-05
Ass1	1.319985	6.77E-07	1.85E-05
Ddx3x	0.888491	6.89E-07	1.87E-05
Ahrr	0.874665	7.17E-07	1.93E-05
Ldhb	1.475139	7.21E-07	1.93E-05
Rab13	0.911104	7.35E-07	1.96E-05
Ifitm6	1.055873	7.39E-07	1.96E-05
Lcn2	3.05954	7.57E-07	2E-05
Cd274	0.906416	7.76E-07	2.02E-05
6330408A02Rik	1.671444	7.92E-07	2.06E-05
Arl3	0.813331	8.88E-07	2.26E-05
C1rl	1.237668	9.24E-07	2.33E-05
Anp32b	1.05848	9.49E-07	2.38E-05
Tnfrsf26	1.043622	9.83E-07	2.44E-05
1600014C10Rik	1.039717	9.91E-07	2.45E-05
Sntb1	3.066097	1.01E-06	2.48E-05
Trim46	2.181907	1.12E-06	2.67E-05
Gm28035	0.812148	1.13E-06	2.7E-05
Adgb	3.80132	1.15E-06	2.73E-05
Gm17082	2.816096	1.21E-06	2.85E-05
Prkar2b	0.93652	1.27E-06	2.96E-05
Aqp9	4.368016	1.31E-06	3.01E-05
Saa3	6.240929	1.33E-06	3.04E-05
Fzd1	3.250641	1.34E-06	3.05E-05
Hp	5.355148	1.35E-06	3.05E-05
Serpinb2	4.598719	1.42E-06	3.21E-05
Bcl2a1d	1.550711	1.44E-06	3.24E-05
Wnk2	3.328589	1.48E-06	3.31E-05
Man2a2	0.897219	1.5E-06	3.33E-05
Nrg1	3.643918	1.5E-06	3.33E-05
F7	1.945618	1.51E-06	3.33E-05
Slc2a6	1.599941	1.53E-06	3.37E-05
Slco3a1	0.95708	1.58E-06	3.45E-05
Ldlr	1.491674	1.69E-06	3.67E-05
Fas	2.307262	1.7E-06	3.69E-05
Fcor	3.89872	1.73E-06	3.72E-05
Sdc1	1.985592	1.79E-06	3.82E-05
Satb1	2.386466	1.79E-06	3.83E-05
Saa1	5.83556	1.86E-06	3.9E-05
Cav1	1.571772	1.9E-06	3.97E-05
Spint1	2.044768	1.97E-06	4.08E-05
Slc39a14	0.906457	2.05E-06	4.21E-05

P2ry13	1.356142	2.13E-06	4.37E-05
Ssh2	1.470897	2.15E-06	4.39E-05
I830077J02Rik	0.973982	2.27E-06	4.6E-05
Ifitm1	1.281166	2.48E-06	4.89E-05
C1ra	0.989559	2.55E-06	5E-05
Pde5a	2.076938	2.58E-06	5.05E-05
Ksr1	1.491388	2.66E-06	5.17E-05
Gm13571	3.137502	3.16E-06	5.93E-05
Stard4	1.039988	3.22E-06	6.02E-05
Gm6560	1.104878	3.4E-06	6.28E-05
Arrdc4	1.092071	3.43E-06	6.3E-05
Gbgt1	1.221639	3.46E-06	6.32E-05
Etv1	1.268976	3.65E-06	6.6E-05
Gbp5	2.206548	3.67E-06	6.62E-05
Gtpbp2	0.869363	3.85E-06	6.86E-05
Il1rl2	4.07699	3.96E-06	7.03E-05
Ift122	1.027001	3.99E-06	7.08E-05
Gypc	1.141636	4E-06	7.09E-05
Fus	1.106026	4E-06	7.09E-05
Arid5b	1.06416	4.03E-06	7.13E-05
Clmp	2.300552	4.12E-06	7.24E-05
Il21r	1.480935	4.34E-06	7.53E-05
Setbp1	3.909909	4.35E-06	7.54E-05
Ccl22	3.169533	4.48E-06	7.74E-05
Epb41l3	2.406531	4.54E-06	7.81E-05
Gbp3	0.932826	4.75E-06	8.11E-05
Plcb1	3.463158	4.79E-06	8.15E-05
Relt	1.248521	4.79E-06	8.15E-05
Tnfsf14	1.725524	4.88E-06	8.26E-05
Serpinb8	1.003728	4.96E-06	8.39E-05
Gm7336	1.100689	5.02E-06	8.45E-05
Clec4n	1.426841	5.11E-06	8.54E-05
Tnfrsf23	0.927297	5.27E-06	8.73E-05
Crocc	0.81679	5.28E-06	8.73E-05
Asrgl1	1.249645	5.36E-06	8.84E-05
Mthfd1l	0.957546	5.7E-06	9.26E-05
Daam2	1.771872	5.77E-06	9.36E-05
Lmo4	1.053668	5.81E-06	9.4E-05
Gm43802	1.041851	5.87E-06	9.44E-05
Gm15886	1.050387	6.03E-06	9.67E-05
Ltb4r1	2.146639	6.49E-06	0.000103
Mgarp	4.134424	6.67E-06	0.000105
Prkce	1.17315	6.74E-06	0.000106

Spry2	1.033814	6.86E-06	0.000108
Slc6a12	2.6679	7.16E-06	0.000111
2210411M09Rik	0.91337	7.96E-06	0.000122
Gm15518	1.117038	8.02E-06	0.000123
Rasgrp1	1.827175	8.07E-06	0.000123
Gm15601	1.309317	8.67E-06	0.00013
Mfsd7a	2.161844	9.13E-06	0.000134
Gm11914	1.74874	9.14E-06	0.000134
Brwd3	0.809073	9.16E-06	0.000134
Jag1	0.933632	9.72E-06	0.000141
Paqr3	0.868459	1.15E-05	0.000163
Gm31166	2.292297	1.16E-05	0.000164
Pf4	1.934166	1.23E-05	0.000171
Slamf6	1.900227	1.27E-05	0.000174
Slc22a4	1.285103	1.28E-05	0.000175
Samsn1	0.915738	1.29E-05	0.000176
Fry	1.070424	1.35E-05	0.000181
Fads2	1.851209	1.47E-05	0.000193
Arsg	1.204585	1.49E-05	0.000195
Jdp2	0.967962	1.51E-05	0.000197
Gm6397	1.995069	1.52E-05	0.000199
Wdr35	1.145868	1.59E-05	0.000206
Cfh	1.165465	1.68E-05	0.000216
Aldoc	1.554396	1.73E-05	0.00022
Tnrc6b	0.832294	1.8E-05	0.000226
Mmp2	1.350761	1.83E-05	0.000229
Gm15987	2.285633	1.89E-05	0.000235
Arid3a	0.870931	2.08E-05	0.000255
Gm8991	1.92242	2.12E-05	0.000259
Gm42809	1.476215	2.24E-05	0.000271
Sort1	0.933993	2.29E-05	0.000276
Vldlr	1.550661	2.38E-05	0.000286
Stat4	1.626273	2.58E-05	0.000305
Acp5	0.878086	2.64E-05	0.00031
Gm26649	0.948154	2.92E-05	0.000337
Hcar2	1.683265	3.27E-05	0.000367
Ciita	0.883049	3.27E-05	0.000367
Slc16a1	0.804592	3.28E-05	0.000368
Mir7676-2	0.815056	3.4E-05	0.000379
Rnf144a	2.162034	3.46E-05	0.000385
Fmnl2	1.072826	3.6E-05	0.000396
Spaca6	1.723264	3.6E-05	0.000396
Itga1	3.457595	3.63E-05	0.000398

Atf5	1.619796	3.64E-05	0.000398
Slc43a3	1.078549	3.67E-05	0.0004
Tnfrsf8	1.139072	3.77E-05	0.000408
Per2	1.100018	3.86E-05	0.000416
Treml2	0.817562	3.92E-05	0.000422
Slc7a11	2.307303	3.98E-05	0.000427
A930007I19Rik	1.858985	4.02E-05	0.00043
Gm5150	0.976779	4.16E-05	0.000443
Bcl2a1a	2.39989	4.19E-05	0.000445
Klf7	1.083848	4.19E-05	0.000445
Ralgps2	0.942666	4.25E-05	0.00045
Zbtb12	1.429835	4.31E-05	0.000455
Gm12960	1.949153	4.47E-05	0.000467
Rnd1	1.627364	4.61E-05	0.000479
Lrrc75a	0.961711	4.73E-05	0.000488
Dcbld2	1.011781	4.82E-05	0.000495
Nemp1	0.846741	4.85E-05	0.000497
Epm2a	1.052023	4.89E-05	0.000499
Gm16096	1.662792	5.03E-05	0.00051
Ddit4	2.318414	5.12E-05	0.000518
Amotl2	2.044326	5.34E-05	0.000538
Chchd10	0.936161	5.36E-05	0.00054
Gm26522	1.680288	5.54E-05	0.000555
Stk26	1.322508	5.64E-05	0.000562
Adamtsl4	0.919414	5.77E-05	0.00057
Ankrd37	1.990433	6.08E-05	0.000597
Eno1b	0.93527	6.18E-05	0.000605
Gm12868	1.507499	6.21E-05	0.000608
Gab1	1.875126	6.4E-05	0.000623
Susd2	2.638471	6.53E-05	0.000631
Ppp1r12b	1.201658	6.57E-05	0.000634
Acta2	1.300622	6.97E-05	0.000667
Gm20547	1.760168	7.07E-05	0.000674
C1rb	1.037955	7.69E-05	0.000721
Ccdc125	1.074906	7.94E-05	0.00074
B430010I23Rik	1.846636	8.04E-05	0.000748
D16Ertd472e	1.488295	8.16E-05	0.000756
Cfb	1.767477	8.5E-05	0.000776
Gm10645	1.059429	8.56E-05	0.00078
Il4	1.019148	8.57E-05	0.00078
Msantd3	2.035244	8.63E-05	0.000784
Ptch1	0.802683	8.66E-05	0.000785
Gm11632	1.007728	9.2E-05	0.000821

Syngap1	1.262092	9.45E-05	0.000841
Rbpms	1.980418	9.45E-05	0.000841
Btg3	0.880926	0.000103	0.000906
Zfyve9	1.107979	0.000104	0.000911
Mir7649	2.286974	0.000108	0.000938
Grhpr	0.952202	0.000115	0.000988
Gm26860	1.214147	0.000122	0.001041
Mthfd2	0.922574	0.000127	0.001073
6530402F18Rik	1.412682	0.000132	0.001109
Cp	1.330216	0.000132	0.001109
Impa2	0.812042	0.000133	0.001112
4930513N10Rik	1.015306	0.000136	0.001134
Il15ra	0.829048	0.000137	0.001135
Gm12070	1.077487	0.000138	0.00114
F13a1	1.529687	0.00014	0.001156
Gstm2	1.65825	0.000144	0.001182
Fam43a	1.30737	0.000149	0.001214
Slc7a2	1.300898	0.000149	0.001215
Nup62	0.908524	0.00015	0.001222
Cep85l	0.812812	0.000155	0.001257
Rbpsuh-rs3	0.894293	0.000156	0.001261
Bach2os	0.954748	0.000159	0.001278
Fam217b	0.840727	0.00016	0.00128
Inpp5j	0.939765	0.00017	0.001348
Ppargc1b	0.934133	0.000174	0.001366
Cd200	1.227901	0.000178	0.001392
Fam161a	0.865489	0.000181	0.001411
Ntng2	1.545116	0.000189	0.00146
Ly75	4.232772	0.000189	0.00146
Kctd1	0.904121	0.00019	0.001467
A430108G06Rik	1.608177	0.000191	0.001467
Gm37468	0.974621	0.000196	0.001499
Svip	0.915955	0.000217	0.001627
Gm19026	1.672821	0.000218	0.001631
Acod1	3.774745	0.000247	0.001811
Ptger2	1.685694	0.000251	0.00183
Gm28913	0.809128	0.000276	0.001987
RP23-228B2.3	0.932016	0.000282	0.002027
Ipcef1	1.820215	0.000301	0.002144
Gm12033	1.051651	0.000303	0.002157
Maml3	0.847422	0.000306	0.002177
Soat2	1.32775	0.000316	0.002228
Mmp9	0.838196	0.000323	0.002269

Ceacam2	0.944961	0.000333	0.00233
Dhcr24	0.980426	0.000338	0.002356
Cd1d1	1.076703	0.00034	0.002373
4930444A19Rik	1.030421	0.000341	0.002378
Il4i1	1.337656	0.000347	0.002411
Gm12537	0.910862	0.000348	0.002414
Glrp1	1.139479	0.000376	0.002586
Ccng2	0.807583	0.000393	0.002683
Igfbp6	1.658865	0.00042	0.002834
Ift80	0.90572	0.000423	0.002853
Gm17041	2.470645	0.000424	0.002857
Cish	2.302062	0.000448	0.002981
Sorl1	0.858052	0.000472	0.003109
Ppbp	2.051117	0.0005	0.003246
Gbp10	1.067312	0.000501	0.003251
Klra2	0.847151	0.000526	0.00339
Spsb4	1.265023	0.000528	0.0034
Casp12	1.530805	0.000556	0.003548
B4galnt2	1.225276	0.000575	0.003645
Gbp6	1.167598	0.000594	0.003746
Gm1840	0.999977	0.000596	0.003754
Malt1	0.861992	0.000599	0.00376
Cebpd	0.900634	0.0006	0.00376
Gm43302	1.174518	0.000619	0.003864
Tmem2	0.802259	0.000638	0.003942
D530033B14Rik	1.522873	0.000643	0.00397
AW112010	1.349125	0.00065	0.004004
Il27	1.079669	0.000667	0.00407
Peli3	0.830798	0.000668	0.00407
Gm11110	1.372153	0.000679	0.004115
Gm26788	1.392722	0.000701	0.004232
Acsl1	1.201362	0.000718	0.004309
Rgs11	1.021694	0.000726	0.004352
Ggn	0.864855	0.000734	0.00438
Ttc26	1.467371	0.000774	0.004586
Mex3b	0.812511	0.000783	0.004631
Amot	0.803326	0.000798	0.004706
Birc3	0.867603	0.000807	0.004752
Pcx	1.061919	0.000822	0.004822
Myo18b	1.454121	0.00083	0.004857
Naip1	0.855417	0.000835	0.004881
Arg1	2.702644	0.000893	0.005176
Ptgir	1.397489	0.000914	0.005272

Tst	0.964617	0.000953	0.005451
Slc39a4	1.101473	0.000985	0.0056
Fstl1	1.337995	0.001001	0.005663
Gm42789	1.053027	0.00107	0.005993
Tmc4	1.142261	0.001115	0.006195
F630040K05Rik	1.698524	0.001205	0.006606
Gm16556	1.333888	0.001228	0.006698
Gm38160	0.853676	0.001277	0.006923
Nedd9	0.809925	0.001298	0.007023
Prelid2	1.015434	0.00134	0.00722
Afap1l1	1.926955	0.001344	0.007232
Mreg	1.236402	0.001358	0.0073
Gm16214	1.165415	0.001494	0.007886
Zfp719	0.813973	0.001499	0.007896
Ccdc138	1.14854	0.001501	0.007901
Tcte3	0.922749	0.001577	0.008207
Kcna3	2.154572	0.001599	0.008291
Mir5131	1.557922	0.001632	0.008446
Gm26756	1.559576	0.001662	0.008578
Tppp3	0.936468	0.001692	0.008701
Clec4e	2.619352	0.001705	0.008746
Tlr2	2.169687	0.001792	0.009125
Mns1	0.867133	0.001969	0.009899
Spata13	2.137192	0.001983	0.009945
Trib3	1.009779	0.002011	0.01005
Mroh2a	0.866935	0.002022	0.01008
Ppm1k	0.811464	0.002037	0.010141
Nfe2	1.364028	0.002077	0.010275
Met	0.93694	0.00219	0.010746
Cyth1	0.952609	0.002216	0.010853
Mir5130	0.875679	0.002249	0.010971
Ppfia3	1.064203	0.002252	0.010975
Gm4117	1.424427	0.002264	0.011017
Ldlrad4	0.877531	0.002279	0.011075
Gm5424	1.006729	0.002316	0.011215
Gm45585	1.282324	0.002343	0.011322
Pgam2	1.130399	0.002368	0.011424
Kif1a	1.357809	0.002387	0.011494
E230032D23Rik	0.876759	0.002396	0.011534
Gm15350	0.861195	0.0024	0.011543
Gm8775	0.852446	0.002402	0.01155
Cxcl3	3.662983	0.002451	0.011741
Il7	1.686274	0.00251	0.011962

Gm44876	1.192046	0.002552	0.012112
Gm10134	1.002226	0.002629	0.012421
Lif	1.914908	0.002752	0.012888
Gm11893	1.223541	0.002877	0.013322
Gm14760	0.857944	0.002885	0.01334
Tex9	0.811749	0.002887	0.013346
Gm10518	0.936523	0.002977	0.013668
Mpzl3	1.095573	0.003013	0.013824
Ssbp2	1.016925	0.00308	0.014069
Gm38342	0.844631	0.00312	0.014222
AI504432	2.129047	0.003176	0.014445
Nfil3	1.983484	0.003243	0.014684
Mir6244	1.32392	0.003278	0.014818
Adora3	1.252738	0.003316	0.014975
Nxpe3	1.528801	0.003322	0.014992
Gm44751	1.124416	0.003502	0.015634
Tubb4a	1.034724	0.003516	0.015685
Gm21370	0.822597	0.003557	0.015797
Pde4d	1.07962	0.003571	0.015841
Gm22643	1.190059	0.003652	0.016129
Gm20658	1.151528	0.004027	0.017448
Rbfox2	1.35121	0.004108	0.017723
Tnfaip2	1.009095	0.004163	0.017937
Gm13408	0.805479	0.004484	0.019048
Sema4c	0.88589	0.004484	0.019048
Slc1a4	1.033731	0.004525	0.019186
Slc25a33	0.84452	0.004616	0.019504
Ikzf2	0.953554	0.004625	0.019525
Ccl5	0.973433	0.004905	0.020504
Chp2	1.195848	0.004949	0.020647
Gm28873	0.873303	0.004971	0.020702
Gm13068	0.937358	0.005074	0.021036
Gm15354	1.049079	0.005242	0.021548
Adhfe1	1.023389	0.005533	0.022487
Ankrd66	1.006931	0.00555	0.022535
Vsig10	1.182136	0.005647	0.02286
Rasgrp2	1.258446	0.005721	0.023115
Sowahc	1.033165	0.005785	0.023317
9530052E02Rik	1.239275	0.00579	0.023328
H2-DMb2	1.06592	0.005931	0.023737
44264	0.888448	0.006033	0.024087
Myo1d	1.18678	0.006278	0.024883
Gm36964	0.931326	0.00645	0.025448

Slc31a2	0.800435	0.006493	0.025572
Gm13803	0.993548	0.006664	0.026078
Gm29233	2.319915	0.006781	0.026417
Zfp473	0.872288	0.006899	0.026804
Gm6652	0.95003	0.007178	0.027757
Gm13568	0.844402	0.007195	0.027781
Ctsc	0.87363	0.007268	0.028001
Gm14023	3.315903	0.007419	0.02849
Vegfa	1.540471	0.007447	0.028571
1700094J05Rik	0.815944	0.00758	0.028978
Thbs1	2.288366	0.00764	0.029171
Ptger3	0.950856	0.007779	0.029601
Prx	1.300773	0.007779	0.029601
Il6	4.27673	0.00799	0.030202
Fam188b	0.951384	0.008417	0.031484
Gm38048	0.921253	0.008551	0.031889
Mir761	1.008709	0.008636	0.032122
Usp27x	1.107239	0.008644	0.032125
Mir6978	0.868533	0.00872	0.032342
Smad6	1.444468	0.008902	0.032856
Rprd1b	0.802874	0.009425	0.034379
Fabp7	0.996708	0.009895	0.035783
Prox2	0.825574	0.010013	0.036141
Amt	1.333143	0.010492	0.037487
Ptgs2os2	1.738709	0.010628	0.037922
Sgk1	1.260242	0.01085	0.038597
G430095P16Rik	1.007824	0.011265	0.039694
Gm12915	0.847895	0.011467	0.040286
Il1a	3.451103	0.011963	0.041627
Gja1	1.907927	0.011971	0.041644
Gm4887	0.941518	0.012515	0.043212
Lmnb1	1.458217	0.012541	0.043272
Gm43592	0.993343	0.012776	0.043824
Il4ra	1.102131	0.012925	0.04423
Runx3	1.677182	0.013426	0.045593
Cd33	0.819842	0.014785	0.049355
Gm27995	0.806111	0.015822	0.051965
Chd7	1.485408	0.015883	0.052062
Gm37563	0.92337	0.015993	0.052331
Loxl2	0.809198	0.016308	0.053082
Gm13033	1.068468	0.01654	0.053652
Gm45010	0.824246	0.016653	0.053949
Ier3	1.230229	0.016811	0.054246

Gm7285	1.058542	0.017655	0.056384
Gm31105	1.157117	0.017894	0.05701
4930483K19Rik	0.824581	0.018786	0.059314
Ptgs2	2.943126	0.019301	0.060541
Gm5803	0.813012	0.01954	0.061174
Ccl2	0.966717	0.019591	0.061244
Gm13292	0.860747	0.020175	0.062759
Uck2	1.561605	0.020284	0.062979
Mir5113	0.888954	0.021437	0.065838
Med12l	1.024975	0.021543	0.066055
Gm4262	0.933746	0.02175	0.0666
Mrgpre	1.11887	0.021974	0.067093
Ahr	1.972982	0.023983	0.071751
Il1b	2.969634	0.024098	0.071938
Gpr84	1.928699	0.02415	0.072069
Cd14	0.812648	0.025036	0.074213
Mir7238	0.838477	0.026102	0.076738
Gm8818	1.444321	0.028773	0.08291
Plk3	1.377346	0.028883	0.083135
Sphk1	0.870333	0.029677	0.084562
Trem1	3.526308	0.029991	0.085234
Gm22240	1.287982	0.031423	0.08847
Olr1	1.737617	0.033258	0.092309
Plekhg6	0.858027	0.033439	0.092712
Flrt3	2.498564	0.036573	0.099523
Osm	1.066556	0.037107	0.100681
Il17ra	1.15673	0.037515	0.101521
Zc3h12c	0.814333	0.03836	0.103308
Gas2	0.822399	0.040362	0.107568
Nr4a2	1.96288	0.042859	0.112524
Gm20412	1.214313	0.046125	0.119265
Nr4a3	2.808786	0.04649	0.120052
4933416M07Rik	1.591631	0.047473	0.122063
Tgm2	1.267294	0.049607	0.12623
Klf4	0.927354	0.049718	0.126439
Nod2	1.048371	0.050116	0.12718
Gpr35	1.29571	0.051988	0.130686
Rab11fip1	1.446986	0.058159	0.142254
Fosl2	1.719709	0.060848	0.146844
Trib1	1.047461	0.080443	0.181653
Ccrl2	1.172735	0.083406	0.186539
Cfap43	1.105091	0.084649	0.18862
0610040F04Rik	0.866138	0.085995	0.190891

Il1r2	2.048137	0.101822	0.21688
Hilpda	0.949272	0.103196	0.219093
Irs2	1.037216	0.107112	0.225573
Adora2b	0.831646	0.114779	0.237005
Nlrp3	1.276995	0.115137	0.237493
Arid5a	0.999591	0.118066	0.241704
Fosb	0.935168	0.134324	0.266749
Nr4a1	0.833012	0.140706	0.276397
Pde4b	1.918108	0.142431	0.278736
Ets2	1.107061	0.156971	0.299034
Gem	1.271843	0.162169	0.306163
Socs3	1.215761	0.166864	0.312502
Plaur	0.978874	0.184111	0.335141
Mir155hg	1.794749	0.194289	0.348231
Marcksl1	1.039837	0.195066	0.34929
Cxcl2	1.16753	0.216237	0.375962
RP24-233B16.1	0.889635	0.226515	0.387687
Cxcl10	0.824607	0.265059	0.431382
Kdm6b	1.045062	0.275887	0.444243
Csrnp1	0.860393	0.279042	0.447998
Kdm6bos	1.005544	0.296248	0.467269
Gm28592	0.950994	0.318821	0.49195
Phlda1	0.941276	0.340569	0.514467
Cxcl1	0.893805	0.482636	0.646899

j. Sequencing result-sig_UP_miRNAs

miRNA	logFC	PValue	FDR
novel_106	12.28233	4.48E-05	0.035612
novel_8	12.35232	0.000189	0.057432
novel_1	9.761721	0.00038	0.072865
novel_36	11.44348	0.000399	0.072865
novel_176	11.08442	0.002005	0.30314
novel_55	13.1364	0.002327	0.30314
novel_15	10.09878	0.003088	0.352074
hsa-miR-6087	6.100289	0.003991	0.404417
novel_215	10.36707	0.006575	0.53165
novel_113	10.16936	0.008825	0.53165
novel_260	8.6301	0.009196	0.53165
novel_362	8.655961	0.009767	0.53165
novel_122	8.545456	0.009993	0.53165
novel_19	4.754513	0.009998	0.53165
novel_24	9.404253	0.011685	0.53165
novel_48	9.122858	0.01223	0.53165
novel_53	9.010074	0.012657	0.53165
novel_22	3.854504	0.01305	0.53165
hsa-miR-4508	4.067717	0.014569	0.53165
novel_384	7.625816	0.015416	0.53165
novel_51	8.341833	0.015619	0.53165
hsa-miR-3656	6.578942	0.016319	0.53165
novel_43	8.3461	0.017908	0.53165
hsa-miR-483-5p	3.406338	0.019784	0.53165
hsa-miR-1224-5p	4.231779	0.020519	0.53165
novel_495	8.102543	0.022244	0.53165
hsa-miR-4516	6.675351	0.02397	0.53165
novel_479	7.710531	0.027275	0.53165
novel_163	7.296266	0.02782	0.53165
novel_496	7.46743	0.031294	0.53165
novel_339	9.263573	0.031315	0.53165
hsa-miR-939-5p	4.039433	0.03328	0.53165
novel_103	6.023141	0.033792	0.53165
novel_490	6.92063	0.033886	0.53165
hsa-miR-92b-5p	3.838484	0.034187	0.53165
novel_454	6.518608	0.037151	0.53165
hsa-miR-320c	2.629316	0.03757	0.53165
hsa-miR-4639-5p	7.536794	0.03809	0.53165
hsa-miR-1228-5p	6.778378	0.038885	0.53165

novel_118	5.67011	0.039555	0.53165
novel_102	6.022878	0.041067	0.53165
novel_301	6.589301	0.041265	0.53165
hsa-miR-320d	2.67328	0.043853	0.53165
hsa-miR-6734-5p	6.657246	0.044941	0.53165
novel_41	2.7902	0.044975	0.53165
hsa-miR-3663-5p	6.253154	0.048305	0.53165
